# A Critical Review of Mycotoxin Contamination in Food and Feed in the Democratic Republic of the Congo and Neighboring Countries: Challenges and Future Directions

**DOI:** 10.3390/toxins18040182

**Published:** 2026-04-10

**Authors:** Michel Kawayidiko Kasongo, Arthur Mpanzu Duki, Christophe Tsobo Masiala, Sarah De Saeger, José Diana Di Mavungu

**Affiliations:** 1 Laboratory of Food Analysis, Faculty of Pharmaceutical Sciences, University of Kinshasa, Kinshasa XI P.O. Box 212, Democratic Republic of the Congo; tkasmich@gmail.com (M.K.K.); arthurduki@gmail.com (A.M.D.); chrismasiala@yahoo.fr (C.T.M.); 2 Centre of Excellence in Mycotoxicology and Public Health, MYTOX-SOUTH^®^ Coordination Unit, Department of Bioanalysis, Faculty of Pharmaceutical Sciences, Ghent University, Ottergemsesteenweg 460, 9000 Ghent, Belgium; sarah.desaeger@ugent.be; 3 Laboratory of Integrative Metabolomics, Department of Translational Physiology, Infectiology and Public Health, Faculty of Veterinary Medicine, Ghent University, Salisburylaan 133, 9820 Merelbeke, Belgium

**Keywords:** mycotoxins, mycotoxin co-occurrence, contamination, foods and feeds, DRC and neighboring countries, cross-border exchanges of mycotoxins, surveillance and control strategies

## Abstract

Mycotoxin contamination remains a persistent threat to food safety in the Democratic Republic of the Congo (DRC) and neighboring countries, driven by conducive tropical agroecological conditions, inadequate post-harvest practices, and limited regulatory governance. This critical narrative review (2009–2024) synthesizes the occurrence data for major staple foods (maize, peanuts, cassava, sorghum, millet, and beans) and dairy products compiled from Google Scholar, ScienceDirect, MDPI and institutional sources. It examines the co-occurrence patterns, exposure pathways, and analytical and regulatory gaps. Warm, humid lowland environments favor Aspergillus and aflatoxins, whereas cooler, humid highland zones promote Fusarium, fumonisins, and deoxynivalenol. Across commodities, contamination intensifies along food value chains through inadequate drying, non-hermetic storage, insect damage, and prolonged handling, with processed products generally exhibiting the highest levels of mycotoxins. Regulated mycotoxins, including aflatoxins, fumonisins, trichothecenes, ochratoxins, and zearalenone, frequently exceed European Union (EU), East African Community (EAC), and Codex Alimentarius Commission (CAC) limits in staple foods. Their co-occurrence is widespread, including emerging mycotoxins such as beauvericin and enniatins, particularly in maize- and peanut-based products, raising concerns about potential additive or synergistic effects. Aflatoxin M1 in milk highlights plant–feed–animal–human transfer within a One Health framework. Despite increasing evidence, the available data remain fragmented and heterogeneous; rapid tests dominate, while few studies employ multi-mycotoxin LC-MS/MS methods. Cross-border trade between countries, such as Uganda, Tanzania, Zambia and Angola, facilitates the circulation of contaminated commodities in the absence of harmonized standards and risk-based controls. Priorities include harmonized regional surveillance, biomarker-based co-exposure assessment, cost-effectiveness evaluation of mitigation strategies, and regulatory alignment at borders. Coordinated, multisectoral action is essential to reduce chronic dietary exposure and improve food safety across the region.

## 1. Introduction

Mycotoxin contamination of food has become an increasing global concern, particularly in regions such as the Democratic Republic of the Congo (DRC) and its neighboring countries, where unique challenges, such as climatic conditions, inadequate storage infrastructure, and limited regulatory enforcement, have exacerbated the issue, not only because of the harmful effects of these compounds on human or animal health [1,2,3,4], but also because of the socio-economic consequences that result [5,6,7]. Mycotoxins are secondary metabolites produced by microscopic fungi capable of colonizing many substrates, such as plants and food products, under certain conditions, in particular appropriate temperature and humidity [3,7,8]. Among the hundreds of identified mycotoxins, the most common and concerning in the DRC and its neighboring countries include aflatoxins, fumonisins, trichothecenes, ochratoxins and zearalenone (ZEN) due to their frequent occurrence in staple foods and their severe health and economic impacts [8,9]. These mycotoxins are subject to regulation under various national, regional or international legislative frameworks [10,11]. In addition to these regulated mycotoxins, investigations are also increasingly focused on emerging mycotoxins, such as beauvericin (BEA), enniatin B (ENN B), 3-acetyl-deoxynivalenol (3-ADON), 15-acetyl-deoxynivalenol (15-ADON), alternariol, and alternariol monomethyl ether (AME) [12,13,14], because of their increasingly frequent appearance in foods [15,16]. Their simultaneous presence could be the basis of antagonistic, additive or synergistic effects [17].

The fungi-generating mycotoxins belong to many genera, such as Aspergillus, Fusarium, Alternaria, Penicillium, and Claviceps [18,19]. They are generally saprophytes and live at the expense of the organic matter that they decompose. Mycotoxin production, the process that leads to the synthesis and excretion of mycotoxins into the environment, depends on several factors that can be intrinsic (genetic) or extrinsic (environmental). Among the intrinsic factors, genetic polymorphism remains the basis of the different characteristics observed in molds [20,21,22]. Environmental factors act in a combined manner on the development and survival of fungi as well as on the production of mycotoxins [23]. They can be physical (temperature, water activity) [24,25,26,27], chemical (pH, gas composition, nature of the substrate, and chemical treatment of the environment) [22,23] or biological (specificity of the species, interaction between microorganisms, presence of mites and insects, and agricultural practices) [3,28,29].

The harmful effects of mycotoxins on human or animal health can be categorized into acute and chronic effects. Acute exposure, resulting from the short-term ingestion of high levels of mycotoxins, can lead to severe symptoms, such as liver failure, immune suppression, and even death, as observed in outbreaks in Kenya and Tanzania in 2004 and 2016, respectively, following the consumption of maize contaminated with aflatoxins [11]. More recently, in June 2024, the Zambian government announced that the country’s market of maize grains and flour for animal feed was contaminated with aflatoxins at high levels [30]. These products caused the death of dozens of dogs according to the Zambian government’s press release; this information was relayed by the government of the DRC, which launched an alert to attract public attention and strengthen surveillance at the borders [31,32]. On the other hand, chronic exposure, which occurs over prolonged periods at lower levels, has been linked to serious health conditions, such as carcinogenicity, estrogenicity, neurotoxicity, genotoxicity, nephrotoxicity, immunotoxicity, hepatotoxicity, and teratogenicity or mutagenicity in humans and animals [33]. Furthermore, global estimates of food crop contamination by mycotoxins, which the Food and Agriculture Organization (FAO) previously estimated at 25%, have shown an increasing trend worldwide [7]. Episodes of contamination undoubtedly lead to socio-economic consequences that have been financially assessed in certain countries, such as the USA [5]. They are frequently the basis for the rejection of many African products that do not comply with EU regulatory limits for aflatoxins, particularly aflatoxin B1 (AFB1) [11].

Given the DRC’s climatic conditions, characterized by consistently high temperatures, excessive humidity, and frequent heavy rainfall, the country faces an increased risk of mycotoxin contamination. These factors create ideal conditions for fungal proliferation during both growing and storage periods, exacerbating food safety challenges. The DRC, in particular, is significantly affected by food contamination caused by mycotoxins. This review provides an updated synthesis of mycotoxin contamination in food commodities in the DRC and its neighboring countries over the period 2009–2024. It examines the occurrence of key mycotoxins, the affected commodities, and their associated health and socio-economic implications. While previous reviews have addressed mycotoxin contamination at the level of individual countries (e.g., Uganda [9]) or regional groupings, such as West and Central Africa [34], Southern Africa [35], and East Africa [36], few studies have adopted an integrated approach encompassing the DRC and all nine of its bordering countries (6.92 million km^2^) [37]. In this context, the present review combines the occurrence data with analysis of agroecological conditions, post-harvest practices, cross-border trade dynamics, and regulatory frameworks, providing a more comprehensive understanding of the drivers that shape mycotoxin contamination across Central Africa.

## 2. Review Methodology

### 2.1. Review Type and Overall Approach

This study is a critical narrative review aimed at synthesizing and analyzing the available data on mycotoxin contamination in food and feed commodities in the DRC and its neighboring countries. Beyond compiling the reported occurrence data, the review adopts a comparative and integrative perspective to identify the overarching trends, key drivers of contamination and major knowledge gaps. Particular emphasis is placed on linking the occurrence data with the agroecological conditions, post-harvest practices, trade dynamics, and regulatory contexts.

### 2.2. Literature Search Strategy

A structured literature search was conducted covering the period 2009–2024 using major scientific databases, including Google Scholar, PubMed, ScienceDirect, and Scopus, as well as regional and institutional sources (e.g., AJOL, FAO, WHO, Codex Alimentarius, CIRAD, and IITA). The search terms included combinations of keywords, such as “mycotoxin”, “aflatoxin”, “fumonisin”, “ochratoxin”, “food contamination”, “feed contamination”, and the names of the DRC and its neighboring countries. Peer-reviewed articles in English and French were considered to capture both the international and regional scientific outputs.

### 2.3. Inclusion and Exclusion Criteria

Studies were eligible if they (i) were conducted in the DRC or its neighboring countries, (ii) were published between 2009 and 2024, and (iii) reported quantitative data on mycotoxin contamination in food or feed. Studies were excluded if they did not clearly specify the geographical origin of samples or fell outside the defined study region.

### 2.4. Data Extraction and Analysis

For each eligible study, the relevant information was extracted, including the country, commodity (matrix), type of mycotoxin, concentration levels, and analytical methods used. The data were organized by commodity and mycotoxin type to enable cross-study comparison. A qualitative assessment of methodological aspects was also performed, considering factors such as the sampling design, analytical technique (e.g., ELISA vs. chromatographic methods), and reported validation parameters. These elements were used to support the critical interpretation of occurrence data and to identify limitations affecting data comparability across studies.

## 3. The Democratic Republic of the Congo (DRC) and Its Border Countries

### 3.1. Geographic and Demographic Overview

The DRC is a vast Central African country covering 2,345,410 km^2^. Located at the heart of the continent, it shares borders with nine countries: Angola (via the Cabinda enclave) and the Republic of Congo to the west; the Central African Republic and South Sudan to the north; Uganda, Rwanda, Burundi and Tanzania to the east; and Zambia and Angola to the south (Supplementary Figure S1). In 2024, the population of the DRC was estimated at 109.3 million inhabitants and that of its neighbors at 229.6 million, including 37.9 million in Angola, 5.3 million in the Central African Republic, 11.9 million in South Sudan, 50 million in Uganda, 14.3 million in Rwanda, 14 million in Burundi, 68.6 million in Tanzania and 21.3 million in Zambia [37]. A total of more than 338.9 million consumers in this sub-regional area face varying levels of mycotoxin exposure, influenced by differences in agricultural practices, storage conditions, regulatory enforcement, and dietary habits across these countries. This exposure poses significant challenges, impacting both food safety and socio-economic stability. The identical microclimates generally observed in the border regions of the DRC, characterized by high humidity levels, persistent rainfall, and warm temperatures, create ideal conditions for the proliferation of molds and subsequent mycotoxin production. These climatic factors, coupled with inadequate drying and storage techniques, substantially increase contamination risks and compromise food safety across the region. Furthermore, they facilitate the cross-border spread of mycotoxins through the trade of contaminated products, as the climatic conditions and agricultural practices in these regions are conducive to mold development and mycotoxinogenesis. The DRC is a member of several regional organizations, such as the Economic Community of Central African States (ECCAS), the Common Market for Eastern and Southern Africa (COMESA), the Southern African Development Community (SADC), the Economic Community of the Great Lakes Countries (ECGLC), the International Conference on the Great Lakes Region (ICGLR) and the East African Community (EAC), all of which are part of the African Union (AU) [38].

### 3.2. Cross-Border Trade

The United States Department of Agriculture (USDA) reported that over the past two decades, maize consumption in the DRC has doubled, rising from 1.1 million tons in 2003 to 2.2 million tons in 2023 [39]. Given the increasing demand driven by a growing population, the country must either expand its industrial agricultural production or continue to rely on imports, which the FAO estimated at a cost of 12 million USD in 2022 [40]. To address its agricultural production deficit and ensure food security for its population [41], the DRC has increasingly turned to neighboring countries, particularly Uganda, Tanzania and Zambia, which are the main exporters in the sub-region. Trade between the DRC and Uganda has grown significantly, with exports, including maize, rising from 479.17 million in 2016 to 687.1 million USD in 2022 [42,43]. In 2023, the DRC accounted for 24.6% of Uganda’s total exports, valued at 695 million USD [44]. Similarly, according to the Tanzanian central bank, the DRC has surpassed Kenya to become Tanzania’s largest export market within the EAC, with exports increasing from USD 170.7 million in 2019 to USD 306.8 million in 2023 [45]. In May 2024, a memorandum of understanding was signed between the DRC and Tanzania to facilitate the export of 500,000 tons of maize [40]. Regarding the trade with Zambia, the DRC shares 2140 km of border, with official trade activity taking place at the key border posts of Kasumbalesa [46] and Kipushi [47], located 90 km and 30 km from the city of Lubumbashi, respectively. An investigation at the Kipushi border post revealed a double barter system, where Congolese traders supply manufactured goods to Zambians in exchange for agricultural products, such as maize, rice, and beans. These products are either consumed directly or stored in Kipushi before being transported to Lubumbashi and Likasi [47]. Meanwhile, the Kasumbalesa post has evolved into a regional hub for international trade flows, particularly for mining and food products. To enhance efficiency and reduce border crossing times, the infrastructure has been modernized with financial support from international donors, including the International Monetary Fund (IMF) and the World Bank [46]. Over the past decade, a market has emerged on the Zambian side of the border, serving as a primary source of cereals and other products destined for major cities in Katanga, such as Lubumbashi, Kolwezi and Likasi [46].

In the south, a 217 km long road is being planned to connect Likasi in the DRC to Solwezi in northern Zambia [48]. Additionally, the Lufu border post between the DRC and Angola is gaining in importance as a key trade route. Located in Kongo Central Province, 80 km south of the city of Matadi and 300 km from the capital Kinshasa, this border post ensures the supply of various commodities from Angola, including maize semolina, rice and dairy products, which are susceptible to mycotoxin contamination [49].

## 4. Occurrence of Mycotoxins in Consumed Foods

This section synthesizes the occurrence of mycotoxins in major food commodities consumed in the DRC and neighboring countries, with a focus on maize, peanuts, cassava, and additional staple foods. Rather than presenting the data by country, the analysis is organized by commodity to enable cross-regional comparison and to identify the consistent contamination patterns, key hotspots, and major drivers of exposure. Particular attention is given to the influence of agroecological conditions, post-harvest practices, supply chain dynamics, and regulatory exceedances, providing a structured framework for interpreting the variability observed across studies.

### 4.1. Maize

Table S1 summarizes the occurrence of mycotoxins in maize across the DRC and neighboring countries. Maize emerges as one of the most highly contaminated commodities in the region, with frequent contamination by aflatoxins and fumonisins, recurrent co-occurrence of multiple mycotoxins, and repeated exceedance of international regulatory limits. These patterns are especially evident in identified hotspot areas, such as Haut-Katanga, Kwilu–Kinshasa, Kivu, Kilosa, Rungwe, southern Zambia, and parts of Uganda and Burundi.

In the DRC, contamination is both widespread and often severe. In the dry tropical savanna of Haut-Katanga, Mwanza et al. [50] reported 100% contamination of maize by fumonisins (2207.5 µg/kg), 95% by total aflatoxin (AFT) (257.5 µg/kg), 45% by ochratoxin A (OTA) (61.9 µg/kg), and 92.5% by ZEN (247.8 µg/kg). In the humid tropical savanna (Kwilu–Kinshasa), Kamika et al. [51] showed a marked increase in contamination along the supply chain, from 32% at the farm level to 100% in market samples, with AFT reaching 560.4 µg/kg, approximately 50 times the CAC regulatory limit of 10 µg/kg. In Kinshasa, Kasongo et al. [52] found that all maize flour samples contained AFT, while 75% contained both DON and NIV; 25% of samples exceeded the EU limits of 2 µg/kg for AFB1 and 4 µg/kg for AFT. The same study also reported emerging mycotoxins (BEA, ENN B, AOH, and AME) and aflatoxins/fumonisins co-occurrences in up to 87.5% of samples. In the humid volcanic highlands of Kivu, Udomkun et al. [53,54] found 100% contamination of grains and flours from households with AFT, with 88% of flour exceeding the EU limit of 4 µg/kg and market levels reaching 320 µg/kg. Probst et al. [55] further reported up to 393 µg/kg of AFT, 9000 µg/kg of fumonisins, and 4000 µg/kg of DON in maize seeds. Matendo et al. [56] showed that processing and storage amplified contamination, with AFT increasing from 3.2 µg/kg in freshly harvested dry maize to 148.9 µg/kg in stored maize flour; all the stored flour samples exceeded the CAC limit of 10 µg/kg.

In Zambia, maize contamination is dominated by fumonisins, although aflatoxin exceedances also occur. Kankolongo et al. [57] found that 21.4% of stored maize grains from the central agroecological zone (valley and forests) were contaminated with aflatoxins with levels up to 108.4 µg/kg, while 96.4% were contaminated with fumonisins, reaching 21,440 µg/kg. Mukanga et al. [58] reported 100% pre-harvest contamination of maize ears, with aflatoxins averaging 5.4 µg/kg in the central zone and fumonisins reaching 73,300 µg/kg in the southern zone. Probst et al. [55] recorded up to 108 µg/kg of AFT and 21,000 µg/kg of fumonisins in maize seeds. Kachapulula et al. [59] showed that in grains, although the AFT levels were lower in the south (12 µg/kg) than in the north (25 µg/kg), exceedances of the CAC limit of 10 µg/kg were more frequent in the south (20% vs. 9%), again indicating the importance of location-specific risk patterns rather than average levels alone.

In Uganda, maize contamination is also substantial and varies across agroecological settings. Probst et al. [55] reported seed concentrations reaching 435 µg/kg for AFT, 19,000 µg/kg for fumonisins, and 8000 µg/kg for DON, with 20% of samples exceeding 100 µg/kg for AFT. In the northern dry savanna, Wokorach et al. [60] found grain contamination by aflatoxins (35%), fumonisins (67%), DON (77%), and OTA (12%), with AFT reaching 99.96 µg/kg. In the humid highlands of southwestern Uganda, Murokore et al. [61] reported that 74.2% of maize flour samples were contaminated with AFT, with a mean of 34.07 µg/kg, and 58% exceeded the EAC limit of 10 µg/kg. In Masindi, in western Uganda, 45% of samples were positive for AFT, with levels up to 45 µg/kg [62]. These results confirm Uganda as an important maize contamination zone, with both dry savanna and humid highland environments contributing to the risk.

In Tanzania, maize contamination shows particularly strong hotspot behavior and high co-occurrence. Probst et al. [55] reported the presence of AFT and fumonisins in seed samples, with mean concentrations of 2 µg/kg and 1000 µg/kg, respectively. In Kilosa, located in the hot, humid region of the eastern lowlands, Kamala et al. [63] measured 1081 µg/kg of AFB1 and 2196 µg/kg of DON in maize grains. In Rungwe, in the very humid southwestern highlands, FB1 and FB2 reached 18,184 and 38,217 µg/kg, respectively. Across the study, up to 73% of samples were contaminated; co-occurrence affected 87% of samples, of which 45% consisted of carcinogenic mycotoxins. In Iringa, Kilimanjaro, and Tabora, infant formula was 100% contaminated with fumonisins and 82% of samples were co-contaminated; six months later, in Tabora, all of the samples contained AFB1 (up to 10.21 µg/kg) and AFT (up to 14.91 µg/kg) [64]. In the northern mountainous areas (Kikelelwa), 32% of infant flours contained aflatoxins, 83% fumonisins, and 44% DON, at levels of up to 386, 2284, and 825 µg/kg, respectively [65]. This trend was confirmed in Rombo, where aflatoxins (58%) and fumonisins (31%) were found in infant flours; more than 48% of samples exceeded the EU limits for AFT (4 µg/kg) and for fumonisins (200 µg/kg) [66]. Boni et al. [67] reported 49.5% grain contamination by AFT (12.48 µg/kg), exceeding the CAC and EU limits by 11%.

In Rwanda, contamination is widespread but generally lower than in the most severe hotspots, although clear regional gradients are visible. Probst et al. [55] detected 300 µg/kg of fumonisins in seeds. Umereweneza et al. [68] showed that 100% of flours were contaminated by AFT, 89% by AFB1, 67% by FB1, and 11% by OTA. The highest levels of AFT were observed in the Eastern Province (Nyagatare: 16.8 µg/kg), and the highest levels of fumonisins in the Southwest Province (Ruhango: 48.1 µg/kg). Niyibituronsa et al. [69] reported 100% contamination of grains by aflatoxins and fumonisins, with an average of 6.69 µg/kg for AFT and a peak in the Western Province (Ruzizi: 13.75 µg/kg). Approximately 10% of samples exceeded 10 µg/kg. Ntwali et al. [70] recorded higher AFT levels in grains from volcanic areas (40.45 µg/kg) than from savannas (4.66 µg/kg).

In Burundi, Udomkun et al. [54] reported 100% contamination of grain and flour in markets, with a maximum of 350 µg/kg of AFT in flours, 70% of which exceeded 4 µg/kg. Nsabiyumva et al. [71], however, found lower contamination at harvest, with 79% of samples contaminated with AFT, but only 3% exceeding the EAC/CAC limit of 10 µg/kg, again pointing to the importance of post-harvest handling and marketing stages in contamination build-up.

The data from other neighboring countries remain limited but still indicate non-negligible risks. In the Republic of Congo, 100% of samples from Brazzaville contained AFB1 (0.4–120.9 µg/kg) [72]. In Angola, Panzo et al. [73] found aflatoxins, fumonisins, OTA, and ZEN in 100% of samples from Luanda, of up to 5929 µg/kg.

Overall, maize contamination in the region is characterized by four main features: (i) the high prevalence of aflatoxins and fumonisins; (ii) the frequent exceedance of EU, CAC, and EAC limits; (iii) strong hotspot effects linked to agroecological conditions in locations such as Haut-Katanga, Kivu, Kilosa, Rungwe, and southern Zambia; and (iv) its substantial amplification during post-harvest handling, storage, processing, and marketing. These patterns confirm maize as a strong driver of chronic dietary exposure to mycotoxins in Central and Eastern Africa and justify its central place in surveillance and mitigation strategies.

### 4.2. Peanuts

Table S2 summarizes the occurrence of mycotoxins in peanuts across the DRC and neighboring countries. Peanuts represent one of the highest-risk commodities for aflatoxin contamination in the region, with consistently high prevalence, frequent extreme concentration levels, and widespread exceedance of international regulatory limits. Distinct contamination hotspots have been observed in locations such as Lubumbashi, Kinshasa, South Kivu, Chipata, Copperbelt, Kampala, Iganga, Babati, and Kicukiro, reflecting strong agroecological and post-harvest influences.

In the DRC, contamination is both widespread and often severe across contrasting environments. In the dry tropical savanna of Lubumbashi, Mwanza et al. [50] reported that 100% of grain samples were contaminated with AFB1 (368.7 µg/kg) and 72.5% with OTA (11.6 µg/kg). In the humid tropical savanna of Kinshasa, Kamika et al. [74] showed that 72% of raw peanuts were contaminated with AFB1 (229.1 µg/kg), with nearly 70% exceeding the EU limit (2 µg/kg), especially during the rainy season. Later, Kamika et al. [75] confirmed this trend by observing 100% contamination by AFB1 (97.4 µg/kg) and AFT (206.2 µg/kg). Kasongo et al. [52] observed 80% contamination of peanut paste by AFB1 and AFT, of which 20% exceeded 2 and 4 µg/kg, respectively, with detection of BEA (225.4 µg/kg). In the volcanic highlands of South Kivu, Udomkun et al. [53,54] reported 100% contamination of both household and market samples, with flours showing the highest levels at markets (mean: 1027.5 µg/kg; max.: 1620 µg/kg), confirming this region as a major hotspot.

In Zambia, the contamination patterns are highly heterogeneous. Bumbangi et al. [76] detected AFB1 (0.45 µg/kg) and AFT (0.43 µg/kg) in raw peanuts from Lusaka, with 12% exceeding the EU limit for AFT (4 µg/kg). In the eastern region, Njoroge et al. [77] reported very high levels of AFB1 in flours, notably in Chipata (mean: 703 µg/kg; maximum: 3000 µg/kg) and in the Copperbelt, where AFB1 reached 11,100 µg/kg. Kachapulula et al. [59] showed that 100% of peanuts in the southern zone had AFT levels exceeding 4 µg/kg, while the central zone had the highest average (90 µg/kg), with a maximum of 361.2 µg/kg, highlighting clear regional hotspots.

In Uganda, peanut contamination is particularly severe and widespread across multiple regions and product types. In the southwest, Kitya et al. [78] detected 100% of grain samples as positive for AFT (11.5 µg/kg), with all exceeding the EU limit (4 µg/kg). In Kampala, Osuret et al. [79] reported aflatoxin levels of up to 940 µg/kg in seeds and 725 µg/kg in paste, far exceeding the US FDA limit (20 µg/kg). Muzoora et al. [80] observed mean AFT levels ranging from 516 µg/kg in the east in grains to 11,732 µg/kg in the north in flours. Other studies have confirmed this trend: 63% and 82% of samples were positive for AFB1 and AFT, respectively, in the central region [81]; 79% for AFT in the north [60]; and the maximum for AFT (1327 µg/kg) was in the Lake Albert region [82]. Akullo et al. [83] reported that AFT was present in all grain samples (100%) from the north and east regions, where a maximum was recorded in Iganga (383.25 µg/kg). Thirty percent (30%) of peanut samples from Soroti, located in the east, were contaminated with AFT (0.052 µg/kg) throughout the supply chain [62]. In Kampala, 76% of flours were contaminated with AFT, with a maximum of 296.4 µg/kg [84]. In the southwest, 100% of flour samples were contaminated with AFT, with 91% exceeding 4 µg/kg [61].

In Tanzania, high contamination levels have also been observed, particularly in the northern regions. Kuhumba et al. [85] reported 100% contamination of flour samples in the north, with maximum concentrations of 60.6 µg/kg for AFB1 and 92.4 µg/kg for AFT. Boni et al. [67] observed that 96.1% of grain samples were positive for AFT and 74.4% for AFB1, with a maximum of 40.31 µg/kg for AFT in Babati, in the north.

In Rwanda, contamination of peanuts is widespread, with Umereweneza et al. [68] reporting 100% of flour samples were positive for AFB1 and AFT, with maximum levels of 66.8 and 126.6 µg/kg, respectively, in Kicukiro (Kigali region). The same study also detected OTA (33%) and FB1 (11%), indicating multi-mycotoxin contamination.

In Burundi, contamination is also widespread and severe at the market level, with 100% of peanut samples testing positive for total aflatoxins and maximum levels reaching 2410 µg/kg in flours [54]. However, at harvest, Nsabiyumva et al. [71] reported that although contamination was present in all areas, only 6% of samples exceeded the CAC limit of 10 µg/kg, again indicating significant post-harvest amplification.

In the Republic of Congo, Bidounga et al. [86] identified the presence of Aspergillus, Penicillium and Fusarium in peanuts stored in Brazzaville, confirming a fungal risk during storage.

Overall, peanut contamination across the region is characterized by: (i) the systematic dominance of aflatoxins; (ii) the frequent occurrence of extremely high concentrations (often >1000 µg/kg); (iii) the widespread exceedance of EU, CAC, and FDA regulatory limits; (iv) well-defined hotspot regions, such as South Kivu, Chipata, Copperbelt, Kampala, and Iganga; and (v) strong amplification during storage, processing, and marketing stages. These findings identify peanuts as a strong contributor to dietary aflatoxin exposure in Central and Eastern Africa, particularly in hotspot regions and along vulnerable segments of the supply chain.

### 4.3. Cassava

Table S3 summarizes the cassava contamination data across the DRC and neighboring countries. Compared to maize and peanuts, cassava generally shows lower levels of aflatoxin contamination, although contamination remains widespread and highly dependent on the processing practices, agroecological conditions, and storage. In addition, cassava exhibits a distinct contamination profile, with a relatively greater prominence of fumonisins, DON, and emerging mycotoxins in some settings. In the DRC, in the humid tropical savanna of Kinshasa, cassava flour showed a moderate aflatoxin contamination of 40% for AFB1 and AFT, and more or less high levels for FB1 (80%), DON (60%), and emerging mycotoxins (BEA, ENN B). However, the levels remained below EU limits for AFB1 (2 µg/kg) and AFT (4 µg/kg) [52]. In the volcanic highlands of South Kivu, Udomkun et al. [53,54] reported 100% of samples (grain, flour, and dried roots) were positive for aflatoxins, with higher concentrations in the dried roots (3.5 µg/kg), 20% of which exceeded 4 µg/kg, highlighting a localized hotspot and the influence of processing and drying conditions. In Zambia, cassava flour and chips were only slightly contaminated with AFT (<1 µg/kg), with the exception of one sample of dried chips reaching 16 µg/kg, above the CAC limit (10 µg/kg) [87], indicating that contamination can still arise under unfavorable post-harvest conditions. In southwestern Uganda, 100% of flour samples were contaminated with AFT (16 µg/kg), with 61% exceeding the CAC limit (4 µg/kg) [78]. Oyesigye et al. [88] reported that 5.2% to 32.2% of flour and chip samples were contaminated with AFB1, AFT, and OTA, with over 93% exceeding the CAC limits. Flours had higher concentrations of AFT (38.9 µg/kg), particularly in the north (59.2 µg/kg). These results indicate strong regional variability and emphasize the role of processing and storage. In Tanzania, low contamination of flours by regulated mycotoxins (<10%) was observed, except for ZEN (38%, 8493 µg/kg). Conversely, a high presence of emerging mycotoxins was reported (BEA, ENN B, AOH, and AME) [89]. A similar pattern was observed in Rwanda, where contamination of flours by regulated mycotoxins was low (<10%), except for ZEN (46.8%, 2826 µg/kg), while BEA (89.6%) and ENN B (30%) were common [89]. Umereweneza et al. [68] reported low levels of AFT (0.1–2.7 µg/kg), but higher levels in Ruhango in the south of the country, indicating a regional hotspot. In Burundi, contamination is widespread but generally occurs at low-to-moderate levels. Udomkun et al. [54] reported 100% contamination of cassava samples (grain, flour, and dried roots) by AFT at low levels. The highest levels were found in dried roots (5.4 µg/kg) and flours (4.6 µg/kg), again suggesting post-harvest amplification.

Overall, cassava contamination across the region is characterized by: (i) generally lower aflatoxin levels compared to maize and peanuts; (ii) localized exceedances of EU and CAC limits in specific regions (e.g., South Kivu, southwestern Uganda, and Ruhango); (iii) the strong influence of processing, drying, and storage conditions; (iv) the greater relative importance of emerging mycotoxins and ZEN in some countries; and (v) clear but less intense hotspot patterns compared to maize and peanuts. These findings position cassava as a comparatively lower-risk commodity in terms of aflatoxin exposure, but one that remains vulnerable under suboptimal post-harvest conditions and requires attention, particularly regarding emerging mycotoxins and localized contamination hotspots.

### 4.4. Other Food Commodities (Sorghum, Soybean, Millet, Beans, Milk, Insects, Fruits, Sunflower, Feed and Feed Ingredients)

Table S4 summarizes mycotoxin contamination across additional staple foods and feed-related matrices. Although generally less studied than maize and peanuts, these commodities reveal diverse contamination profiles, including frequent aflatoxin occurrence, localized exceedances of regulatory limits, and important links to both direct human exposure and indirect exposure through the food chain.

Among the secondary cereals and legumes, sorghum, millet, beans, and soybean have shown widespread but variable contamination. Sorghum has been consistently affected across multiple regions. In the volcanic highlands of Kivu (DRC), 100% of flour and grain sorghum samples were contaminated with aflatoxins (4.1–4.9 µg/kg), with 63 to 75% exceeding the EU limit (4 µg/kg) [54]. In Uganda, similar contamination patterns were observed in both the dry savanna of the north and the humid highlands of the southwest, with 66 to 100% of samples (grain, flour) contaminated (10–15 µg/kg), and with 58 to 77% exceeding the EU limit [60,61,78]. In Burundi, the average level of AFT in sorghum (grain, sprouted grain, and flour) ranged from 6.1 to 7.1 µg/kg, with a maximum of 490 µg/kg, indicating occasional extreme contamination [54].

Millet has shown comparable variability, particularly in Uganda, where 21–100% of flour samples have been contaminated with aflatoxins (1.5–14 µg/kg), and 12–91% of positive samples have exceeded the EU limit of 4 µg/kg [60,61,78,90]. Beans display a distinct pattern characterized by multi-mycotoxin co-occurrence. In the dry tropical savanna of Lubumbashi (DRC), 80% of samples contained aflatoxins (mean: 215.5 µg/kg), alongside high prevalence of fumonisins (83.3%), OTA (82.4%), and ZEN (90%) [50]. In the Kivu highlands, 100% of samples were contaminated with aflatoxins (3.5 µg/kg), with 20% exceeding the EU limit of 4 µg/kg, while in Burundi, 100% of samples were positive, with 33% exceeding 4 µg/kg [54].

Soybeans are an expanding crop and present a more contrasting profile. In the highlands of South Kivu (DRC), 100% of samples were positive for AFT (3.7–4.1 µg/kg), with half exceeding the EU limit [54]. In contrast, AFT contamination remained low in the dry savanna of northern Uganda (12.7%; 0.65–1.19 µg/kg) [90] and marginal in Rwanda (3%, only one exceedance) [91]. In Burundi, it was widespread: 100% of samples were contaminated (3.4–6.9 µg/kg), with 12 to 80% of samples exceeding the standard [54], again highlighting the strong regional contrasts.

Animal-derived products reveal a critical exposure pathway. Milk is frequently contaminated with aflatoxin M1 (AFM1), reflecting carry-over from contaminated feed. In the highlands of South Kivu in the DRC, 100% of samples contained it (4.8–261 µg/kg), of which 40% exceeded the EU limit (0.05 µg/kg) [54]. In Burundi, 100% of milk and dairy product samples were positive (8.2–82.8 µg/kg), with 37.5% exceeding the EU limit of 4 µg/kg [54]. These findings highlight the importance of feed contamination in shaping human exposure through animal products.

Non-conventional food sources also contribute to exposure in specific contexts. In Zambia, edible insects collected from urban markets showed notable contamination, with termites exhibiting mean aflatoxin levels of 24 µg/kg; 100% of termite samples and more than 40% of caterpillar samples exceeded the national aflatoxin limit of 10 µg/kg [92]. Similarly, dried wild fruits (*T. garckeana*, *V. lanciflora*, and *S. rautanenii*) showed mean aflatoxin levels of 11, 12, and 57 µg/kg, respectively, exceeding the national regulatory limit of 10 µg/kg [93].

Oil crops and derived products also represent an important contamination pathway. In Tanzania, sunflower samples from Dodoma and Morogoro showed AFT contamination rates of 50 to 71% in seeds (1.7–662.7 µg/kg) and 100% in oil cakes (1.9–536 µg/kg), with 57 to 80% exceeding the US FDA limit of 20 µg/kg [94]. In Singida, seed contamination was lower (15%), whereas unrefined oils remained highly contaminated (80.9%). Moreover, 50% of positive seed samples and 17.65% of oil samples exceeded 2 µg/kg [95].

Finally, feeds and feed ingredients constitute a major indirect exposure route. In Rwanda, samples collected along the feed supply chain were contaminated with aflatoxins and fumonisins, with the mean concentrations exceeding 88 µg/kg and 1000 µg/kg, respectively. More than 85% of AFB1-positive samples from dairy farmers exceeded the national regulatory limit of 5 µg/kg for bovine feed [96], underscoring the link between feed contamination and subsequent AFM1 occurrence in milk.

Overall, these additional commodities highlight: (i) the widespread but heterogeneous contamination across secondary crops; (ii) the frequent exceedance of regulatory limits in both plant and animal-derived foods; (iii) the significant contribution of feed contamination to human exposure via milk; (iv) the presence of contamination in non-conventional foods, such as insects and wild fruits; and (v) strong regional variability linked to agroecological conditions and post-harvest practices.

Together, these findings demonstrate that mycotoxin exposure in the region is not limited to major staples such as maize and peanuts, but extends across a wide range of food and feed matrices, reinforcing the need for integrated surveillance across the entire food system.

**Figure 1:**
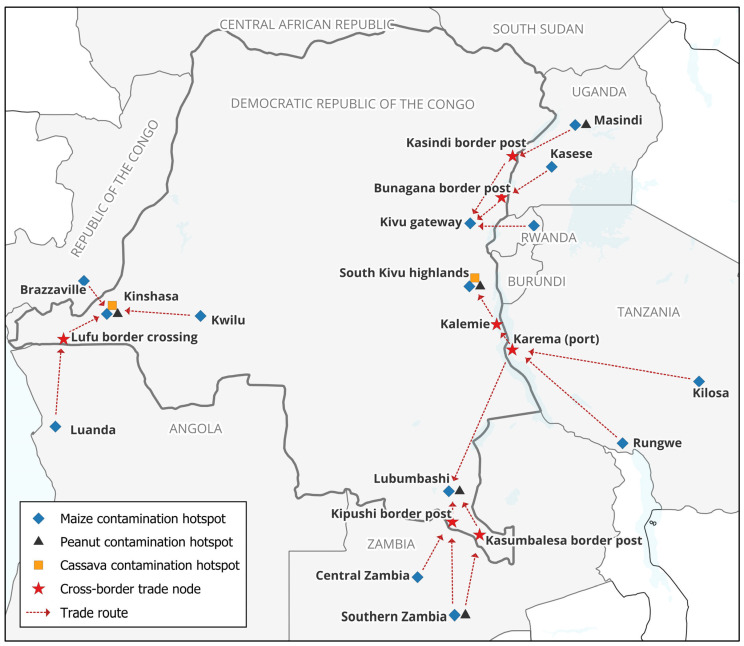
Spatial distribution of major mycotoxin contamination hotspots and indicative cross-border trade routes in the Democratic Republic of the Congo (DRC) and neighboring countries.

The spatial distribution of key contamination hotspots identified across commodities, including major production and trade corridors, is synthesized in Figure 1, which provides an integrated view of contamination patterns and regional connectivity.

The hotspots are represented by commodity-specific symbols (maize, peanuts, and cassava) based on the reported occurrence data. Two distinct high-risk zones are highlighted in the eastern DRC: (i) the South Kivu highlands, characterized by consistently high contamination levels in staple foods; and (ii) the Kivu gateway, representing a key northern interface connecting cross-border trade flows from Uganda and Rwanda into the eastern DRC.

The major cross-border trade nodes (red stars) include Kasindi, Bunagana, Kipushi, Kasumbalesa, and the Lufu border crossing, illustrating the key entry points into the DRC. Additional transport and redistribution nodes include Kalemie (DRC) and Karema (Tanzania port), which together form a key lacustrine corridor across Lake Tanganyika, linking Tanzanian production zones to the eastern DRC.

The trade routes (dashed arrows) depict the indicative flows linking contamination hotspots and production zones across the region, including:

- Uganda (Masindi, Kasese) → eastern DRC via Kasindi and Bunagana;
- Tanzania (Kilosa, Rungwe) → Lake Tanganyika corridor (Karema–Kalemie) → eastern DRC (Kivu highlands) and southern DRC (Lubumbashi);
- Zambia (central and southern regions) → southern DRC (Lubumbashi) via Kasumbalesa and Kipushi;
- Angola (Luanda) → western DRC (Kinshasa) via the Lufu corridor;
- Internal redistribution along the Kwilu–Kinshasa corridor, highlighting the movement of contaminated commodities between production areas and major urban consumption centers.

These routes are conceptual representations derived from published occurrence data and reported trade linkages, intended to illustrate the potential pathways for the regional dissemination of contaminated commodities rather than exact logistical routes. The figure was produced using QGIS based on publicly available geographic boundary data (GADM and Natural Earth) and information synthesized from the reviewed literature. This figure provides a qualitative synthesis of published occurrence data and regional trade dynamics and is intended for conceptual interpretation rather than quantitative risk assessment.

## 5. Socio-Economic Risks and Impacts

Unlike developed countries, most sub-Saharan African nations lack comprehensive data to accurately assess the risks and socio-economic impacts of dietary exposure to mycotoxins through food. Nonetheless, countries such as Tanzania, Uganda and Rwanda have reported cases of acute and chronic poisoning, primarily associated with maize and peanuts, food that are widely consumed in the region.

### 5.1. Impact on Human and Animal Health

In Tanzania, a study by Kimanya et al. [97] on 12-month-old infants consuming maize contaminated with fumonisins at daily doses ranging from 0.003 to 28.838 mg/kg body weight per day found that these children were, on average, 1.3 cm shorter and weighed 328 g less than their peers. Another study conducted on 41 children aged 18 to 24 months from Kikelelwa, who consumed maize-based supplements, revealed that 32% were exposed to aflatoxins (1–786 ng/kg bw/day), 66% to DON (0.38–8.87 μg/kg bw/day) and 56% to fumonisins (0.19–26.37 μg/kg bw/day) above the tolerable daily intake levels. This study further indicated that 10%, 29% and 41% of children were co-exposed to aflatoxins/deoxynivalenol, aflatoxins/fumonisins and fumonisins/deoxynivalenol, respectively [65]. Magoha et al. [66] established a weak but significant inverse association between exposure to AFM1 (1.13 to 66.79 ng/kg bw/day) and growth disorders in infants under six months of age living in Rombo, after analyzing breast milk samples at 1, 3 and 5 months. Subsequent analyses of maize-based foods consumed by infants under six months indicated that daily intakes of aflatoxins (0.14 to 120 ng/kg bw/day) significantly exceeded the European Food Safety Authority (EFSA) limit of 0.017 ng/kg bw/day [98]. Analyses carried out on 166 infants at birth, six months and twelve months established an association between blood biomarkers of aflatoxin (aflatoxin–albumin adducts, AFB-Alb), urinary biomarkers of fumonisin B1 (UBF1) and growth retardation. At birth, the infants exhibited a growth retardation of 44%, associated with respective concentrations of AFB-Alb and UBF1 of 4.7 pg/mg of albumin and 313.9 pg/mL of urine. At six months, their growth retardation increased to 55%, with corresponding levels of 12.9 pg/mg of albumin and 167.3 pg/mL of urine. By 12 months, their growth retardation reached 56%, associated with respective doses of 23.5 pg/mg of albumin and 569.5 pg/mL of urine [99]. Children aged 6 to 12 months consuming maize-based flours were exposed to daily doses six, three and two times higher than the authorized limits for aflatoxins (0.017 ng/kg bw/day), fumonisins (2 μg/kg bw/day) and DON (1 μg/kg bw/day), respectively [100]. Chen et al. [101] found that 80% of urine samples collected from 94 children aged 24 to 36 months in Haydon contained UBF1 at a level of 1.3 ng/mL of urine, establishing a significant association between dietary fumonisin exposure and observed underweight. In 2016, 20 out of 68 individuals poisoned in the center of the country died after consuming maize contaminated with aflatoxin and fumonisin at concentrations reaching 51,000 and 12,630 µg/kg, respectively [102]. In 2017, in the Kiteto district in northeastern Tanzania, four deaths were recorded out of eight cases of aflatoxin poisoning [103].

In Uganda, Asiki et al. [104] detected AFB-Alb in 100% of adult serum samples (0–237.7 pg/mg albumin) and in 96% of child serum samples. Similarly, Kang et al. [105] analyzed serum samples from residents of the southwest of the country, and found aflatoxin-lysine adducts (AFB1-Lys) in 642 out of 713 samples at levels ranging from 0.40 to 168 pg/mg albumin. Wokorach et al. [60] demonstrated that consumption of sorghum grains exposed adults to fumonisins at levels eight times more and infants 46 times higher than the tolerable daily intake (TDI). The exposure of adults and children to fumonisins through maize was six and 33 times higher than the TDI, respectively. Compared to millet and peanuts, sorghum and maize grains posed a greater risk of exposure to aflatoxins, OTA and DON. The risk was further exacerbated by the co-occurrence of multiple mycotoxins, observed in 40.7% of samples. Kitya et al. [78] reported a link between exposure to aflatoxins and the incidence of hepatocellular carcinomas, which was associated with the consumption of staple foods such as maize, sorghum, cassava and peanuts. The risk was particularly high in the northern region due to the high prevalence of aflatoxins in foods and a hepatitis B virus incidence rate of 17.6% [106]. In a cohort study involving 220 mother–infant pairs, Lauer et al. [107] found that 100% of serum samples from pregnant mothers tested positive for AFB1–lysine (AFB-Lys) adducts at levels ranging from 0.71 to 95.60 pg/mg albumin. The study established a significant association between aflatoxin exposure during pregnancy and growth retardation in newborns, including low birth weight and smaller head circumference. Atukwase et al. [84] estimated that aflatoxin intake from maize and peanut products among mothers (15–44 years) and children (6–59 months) in Kampala ranged from 0.01 to 0.91 µg/kg bw/day, exceeding the EFSA’s TDI of 0.017 µg/kg bw/day. This exposure was associated with an estimated risk of developing primary liver cancer at rates of 7.6 and 5.4 cases per 100,000 persons per year for mothers and children, respectively. A risk assessment of AFB1 exposure through cassava consumption revealed that populations in eastern Uganda (8.29 ng of AFB1/kg bw/day) had a lower risk (0.67) compared to those in the northern region, where a daily dose of 67.5 ng of AFB1/kg bw/day corresponded to a higher risk (3.44) of developing hepatocellular carcinoma [88].

In Rwanda, Collins et al. [108] observed that women of childbearing age (*n* = 139) were exposed to several mycotoxins above their TDI. A plasma analysis revealed AFB-Lys adducts in 81% of samples, while a urine analysis detected ZEN, DON, FB and OTA in 61, 77, 30 and 71% of samples, respectively.

### 5.2. Economic Impact

Mycotoxin contamination of crops poses a significant threat to food security and economies worldwide [109]. The presence of mycotoxins in food beyond the maximum limits leads to substantial losses, not only in terms of food waste but also in economic revenues for both governments and populations. Global crop contamination rates are estimated to be increasing [9], currently ranging between 62 and 80% [7,52]. This results in losses of billions of metric tons of contaminated food annually, with Africa being particularly affected. Approximately 60% of the African population relies primarily on subsistence farming [110]. In Kenya, more than 2.3 million bags of maize were destroyed due to aflatoxicosis between 2004 and 2006. Strict regulations in developed countries, especially within the EU, a major importer of African products, have further restricted exports, leading to annual losses amounting to several million US dollars per year. Since 2000, numerous African exports have been rejected at EU borders due to mycotoxin contamination [9]. Countries such as Egypt, Nigeria, Ghana, Morocco, Tunisia and Rwanda have faced such challenges. For instance, Rwanda’s maize, sorghum and soya exports were denied entry into the United Kingdom due to aflatoxin contamination [6]. Similarly, in 2013, the World Food Program (WFP) rejected 60,000 bags of Kenyan maize, and in 2019, Kenya recalled five brands of maize flour from stores due to aflatoxin contamination [103]. These issues have contributed to an estimated annual loss of approximately 450 million US dollars for African countries [6]. Beyond the direct costs associated with losses and export rejections, mycotoxins also incur significant healthcare expenses due to the diseases they cause. In sub-Saharan African countries, these economic burdens are challenging to quantify accurately due to the limited statistical data. However, countries like Tanzania, Uganda and Rwanda have provided insights. Despite being among the top 25 maize producers worldwide and one of the largest in sub-Saharan Africa, Tanzania loses nearly 30 to 40% of its post-harvest production annually due to mycotoxin contamination [111]. In Uganda, Lukwago et al. [9] reported that aflatoxin contamination contributed to a reduction in economic growth by 0.26%, a 43.5% decline in overall exports and a 45% decrease in cereal exports. These losses translated into reductions in household income and consumption by USD 79.3 million and USD 59.1 million, respectively. Furthermore, export rejections cost Uganda an estimated USD 38 million annually, while the government allocates around USD 577 million to treat approximately 3700 liver cancer patients linked to aflatoxin exposure [9].

## 6. Discussion

### 6.1. Major Food Commodities and Mycotoxins Associated with Contamination

Across the DRC and neighboring countries, mycotoxin contamination is strongly concentrated in a limited number of staple commodities, with maize and peanuts consistently emerging as the primary drivers of exposure both in terms of contamination levels and frequency of regulatory exceedances. This reflects a combination of their high intrinsic susceptibility to fungal infection, favorable agroecological conditions, and their central role in local diets [112]. It should be noted that during the study period, some countries distinguished themselves in the analysis of particular foods. These are Zambia in the analysis of edible insects, including termites [92], and dried fruits [93]; Tanzania in sunflower seeds and their unrefined oils [94,95]; and Rwanda in poultry feed [96]. In the DRC, basic foods, such as rice or dried or smoked fish, likely to be contaminated by mycotoxins were not subject to investigation during this period.

With respect to mycotoxin types, aflatoxins, fumonisins, DON, and ZEN are consistently the most investigated and most widespread across commodities. This pattern aligns with global observations, including the survey by Gruber-Dorminger et al. [16], which identified these compounds as the dominant mycotoxins in samples from sub-Saharan Africa. Furthermore, this review also indicated the low occurrence of mycotoxins such as BEA and ENN B, demonstrating the lack of attention paid to emerging mycotoxins. Indeed, there is currently insufficient evidence based on in vivo toxicity studies implicating emerging mycotoxins [113].

Geographically, the most severe contamination patterns for maize and peanuts are consistently reported in Uganda and Tanzania, where concentrations of aflatoxins and fumonisins frequently exceed the EAC standards. These results are consistent with data from the Partnership for Aflatoxin Control in Africa (PACA), which show high proportions of non-standard samples recorded in different districts of Uganda (Mubende and Kamwenge, Soroti and Iganga) and Tanzania (Morogoro, Tabora and Geita) [9,114,115]. Indeed, poor agricultural practices; poor management of post-harvest crop handling; and non-enforcement of regulations, especially for foods intended for local marketing and consumption, may explain this contamination [116].

### 6.2. Mycotoxin Contamination in the DRC and Neighboring Countries: Key Contributing Factors

The climatic conditions, particularly high temperatures, excessive humidity, and abundant rainfall, together with inappropriate post-harvest practices across these countries, constitute the key determinants that predispose foods to mycotoxin contamination [6].

#### 6.2.1. Influence of Agroecological Zones

The influence of agroecological zones on mycotoxin contamination is critical, as temperature, humidity, rainfall, and pedoclimatic conditions directly determine the proliferation of mycotoxin-producing fungi [117]. In general, warm and humid tropical savannas and equatorial zones favor *A. flavus*, the main producer of AFB1 (optimum ~30 °C), whereas cooler and moist highland environments are more conducive to the Fusarium species responsible for fumonisins and DON (optimum 20–25 °C) [118]. Although this climatic gradient generally holds across crops, the intensity and profile of contamination vary by commodity, intrinsic composition, stress exposure, and post-harvest practices. In maize, the highest AFT levels have been reported in the warm humid lowlands, including the wet tropical savanna of Kwilu–Kinshasa (DRC) [51], the eastern lowlands of Kilosa (Tanzania) [63], and eastern Rwanda (Nyagatare) [68], where Aspergillus thrives. Conversely, the cool, humid volcanic highlands of the Kivu region (DRC) and western Rwanda [55,68] show a predominance of Fusarium toxins, such as fumonisins and DON. Urban processed flours display more complex toxin profiles than grains, reflecting cumulative contamination along the value chain [117]. In humid regions, contamination consistently increases from the field to marketplaces, underscoring the critical role of post-harvest handling [51,83]. In peanuts, the agroecological influence is particularly pronounced due to the lipid-rich matrix, which strongly favors Aspergillus growth. Hot and humid savannas, such as Lubumbashi (DRC) [50], northern Zambia [77], northern/eastern Uganda [79,80,81,82] and northern Tanzania [67,85], exhibit the highest aflatoxin burdens, including in flours and pastes. These trends are consistent across the region, notably in Zambia and Uganda [83,119]. In humid tropical zones (Kinshasa, Kigali), persistent moisture slows pod drying and maintains conditions ideal for fungal growth. Even in the highlands (South Kivu, volcanic zones), frequent orographic rainfall maintains elevated humidity, sustaining the contamination risk, especially in processed products [54]. In cassava, the agroecological influence is more moderate due to its low lipid content, which limits fungal development, and shows aflatoxin synthesis patterns similar to those also observed elsewhere in Africa [52,54,89] and in Brazil [120]. However, in humid savannas and high rainfall zones (Kinshasa, South Kivu), moisture during the growth and drying of chips promotes aflatoxins, fumonisins, and emerging mycotoxins [52,54,55]. In semi-arid regions (Zambia), contamination remains low unless poor post-harvest practices offset the climatic advantages. Other commodities (sorghum, millet, soybean, beans, and milk) also show contamination patterns tightly linked to agroecological conditions. Sorghum and millet, cultivated in dry savannas and humid highlands, often exhibit elevated aflatoxins when alternating humidity and drought induce water stress favorable to Aspergillus. These findings align with PACA reports showing that 90 to 100% of sorghum samples in the dry Ugandan savannas contained AFT, with more than 90% exceeding 10 µg/kg [114]. Soybean contamination remains low in cool climates (Rwanda) but increases in humid zones with artisanal storage (South Kivu, Burundi) [54]. Beans are consistently vulnerable even in cool–moist climates, with up to 100% contamination in the Kivu highlands [54]. In milk, AFM1 reflects upstream exposure to AFB1-contaminated feed, particularly in the humid highlands of Kivu and western Uganda [9].

#### 6.2.2. Impact of the Supply Chain

Mycotoxin contamination generally intensifies along the supply chain, from farm-level production to urban markets. This pattern is clearly illustrated by several studies discussed in Section 4, where the contamination levels measured in market samples (e.g., maize and peanut products in Kinshasa, Kampala, and South Kivu) were substantially higher than those observed at harvest. Inadequate drying on the ground, repeated handling, moisture reabsorption during transport, non-airtight storage, and extended holding times collectively promote fungal proliferation [83]. Post-harvest steps thus represent a critical control point, with variable effects depending on the commodity and degree of processing. For maize, contamination clearly increases from farm to market. In the humid tropical savanna of Kwilu–Kinshasa (DRC), contamination rose from 32% at the farm level to 100% in the market samples, with aflatoxin levels reaching 560.4 µg/kg, far exceeding the CAC limit of 10 µg/kg [51]. Insufficient drying, non-hermetic storage, and insect infestation promote fungal colonization, particularly in processed products or those stored for several months [64,121]. For peanuts, the processing steps substantially heighten the risk: ground drying, shelling, and milling increase moisture uptake and surface exposure [83]. Urban lots, often mixed and repeatedly handled, are particularly vulnerable. This is consistent with the extremely high aflatoxin levels reported in market products, such as flours and pastes, in Kinshasa, Kampala, and South Kivu, where concentrations frequently reached several hundred to thousands of µg/kg (Section 4). For cassava, contamination is consistently higher in processed products than in fresh roots [88,122]. Fragmentation, ground drying, rehydration, and artisanal processing facilitate recontamination, particularly in local units and open markets [9,53]. This pattern is especially evident in humid environments such as Kinshasa and South Kivu, where drying conditions are often suboptimal and contamination persists or increases during processing. For sorghum, millet, soybeans, and beans, artisanal milling and uncontrolled storage conditions exacerbate contamination [54]. These effects are particularly relevant in regions where fluctuating humidity and limited storage infrastructure promote fungal development, as discussed in Section 4. For milk, the critical point lies upstream, where contaminated feed directly determines AFM1 levels, irrespective of dairy processing methods [9,96,123]. This is particularly important in humid highland regions such as Kivu and western Uganda, where high levels of feed contamination have been reported, leading to frequent exceedance of the EU limit of 0.05 µg/kg for AFM1 in milk (Section 4).

### 6.3. Cross-Border Trade and Its Role in Mycotoxin Spread

Given the possibility of increased commercial exchanges, the likelihood of cross-border exchanges of mycotoxins poses significant risks to human and animal health, economic livelihoods, and trade. Several studies reviewed in this paper substantiate these concerns. For example, maize produced in bordering countries has been found to contain mycotoxins, such as aflatoxins and fumonisins [61,67]. Research conducted in Uganda on various food products, including maize and groundnuts from agroecological zones bordering the DRC, such as Masindi, Kasese, Mubende, Kamwenge, Kabale and the Lake Albert area, have indicated aflatoxin contamination levels exceeding 50%, with 30 to 100% of samples surpassing the regulatory limit of 10 µg/kg [61,112,114]. These findings suggest that contaminated commodities can circulate across borders and contribute to the persistence and spread of contamination within the region. To mitigate these risks, it is crucial to establish and implement harmonized policies for mycotoxin control and monitoring across the DRC and neighboring countries.

The Democratic Republic of the Congo’s membership in several regional organizations, such as COMESA, ECCAS, EAC, and SADC, represents a strategic asset for the implementation of common policies on health and food safety related to mycotoxins. These organizations can provide privileged platforms to establish early-warning systems, harmonize regulations, and promote cross-border cooperation. They can also play a key role in strengthening the institutional and technical capacities required for effective risk management related to mycotoxins. This regional dynamic has taken on an even more significant dimension at the continental level with the recent creation of the African Food Safety Agency (AFSA) by the AU. During its 38th Ordinary Session held in February 2025 in Addis Ababa, the Conference of Heads of State and Government adopted the statutes of this technical body [124]. Among its missions, the AFSA will be tasked with developing a continental regulatory framework on food safety, providing scientific expertise on and risk assessments of public health threats, strengthening the capacities of African laboratories (contaminant analyses, mycotoxins, chemical residues, etc.), ensuring training and technical cooperation among Member States, and establishing harmonized systems for monitoring contaminants and foodborne diseases [125].

The cross-border trade in mycotoxins will increase further with regional integration. Indeed, the DRC’s accession to the EAC has significantly boosted regional trade, expanding its market opportunities for various products, including maize. Due to its growing population and export-oriented economy, the dynamics of maize exports, once favoring Kenya, are now shifting toward the DRC. Consequently, like Tanzania, Zambia plans to export 20,000 tons of maize to the DRC in the near future [40,126]. At the same time, infrastructure development is accelerating trade connectivity. Uganda is investing in the construction of key roads in the northern DRC, linking Kasindi in Uganda to Beni (80 km), Beni to Butembo (54 km), and Bunagana to Goma via Rutshuru (89 km) [127]. Tanzania is modernizing the port of Karema on Lake Tanganyika to facilitate the movement of goods from the port of Dar es Salaam to Lubumbashi and the eastern DRC via the port of Kalemie on Lake Tanganyika [128].

These expanding trade networks, which will connect several contamination hotspots identified in this review (e.g., eastern DRC, western Uganda, northern Tanzania, and Zambia), are likely to facilitate the regional circulation of contaminated commodities. The spatial overlap of major trade routes and contamination hotspots (Figure 1) further supports this interpretation. This underscores the need for coordinated cross-border surveillance, harmonized regulatory enforcement, and integration of mycotoxin control measures within regional trade systems.

### 6.4. Co-Occurrence of Mycotoxins in Food Commodities

The co-occurrence of mycotoxins is widespread across the region and represents an underestimated cumulative risk. Resulting from simultaneous or sequential contamination events along the supply chain, co-exposure constitutes a major public health concern because the combined toxic effects may be additive, synergistic, or amplified beyond those of individual toxins [52]. Maize exhibited the highest frequency of co-occurrence: more than 50% of samples contained at least two mycotoxins, reaching 80 to 90% in some areas, particularly in hotspot regions such as Kivu (DRC) and Tanzania (e.g., Kilosa and Rungwe), described in Section 4. The dominant combinations involved aflatoxins and fumonisins, sometimes accompanied by OTA, thereby enhancing the hepatocarcinogenic, nephrotoxic, neurotoxic, and immunotoxic effects [33]. In peanuts, aflatoxins were predominant but frequently co-occurred with OTA, fumonisins, or emerging toxins such as BEA, particularly in flours and pastes, where multi-mycotoxin analytical approaches readily detected complex patterns. This is consistent with the findings from Kinshasa, Kampala, and South Kivu, where highly processed peanut products showed both elevated concentrations and multi-mycotoxin profiles (Section 4). In cassava, aflatoxin levels were generally low; however, processed products often contained fumonisins, DON, NIV, OTA, and multiple emerging mycotoxins (BEA, ENN B, AOH, and AME), forming a discreet yet relevant source of combined exposure, particularly in humid environments such as Kinshasa and South Kivu [52,53,54]. Other commodities also showed co-occurrence, although less consistently documented. Beans were particularly affected, with frequent combinations of AFT, FB, OTA and ZEN, especially in regions such as Lubumbashi and Kivu (Section 4). In animal-derived products, AFM1 in milk originating from AFB1-contaminated feed may contribute to a “cocktail effect,” especially in children.

Overall, the widespread co-occurrence of mycotoxins across multiple commodities highlights the limitations of single-toxin risk assessment approaches and underscores the need for multi-mycotoxin monitoring strategies and integrated risk assessment frameworks adapted to regional exposure patterns.

### 6.5. Exceedance of International Regulatory Limits

A regional analysis indicates that several staple foods regularly exceed CAC and EU regulatory limits, occasionally by large margins. This non-compliance is particularly concerning for infants, who consume maize, cassava, or sorghum-based porridges and are therefore vulnerable to chronic and cumulative exposure. Regulatory exceedances reflect serious deficiencies in post-harvest handling and quality control, representing a major public health challenge. Maize displayed the most critical exceedances: in the DRC, AFB1 reached 560 µg/kg (280-fold higher than the EU limit), whereas fumonisins in Tanzania exceeded 38,000 µg/kg. Even in Rwanda, where control measures are stronger, approximately 10% of samples remained non-compliant, exposing populations to increased risks of hepatocellular carcinoma, stunting, and immunosuppression [33]. In peanuts, exceedances were widespread, especially in artisanal flours and pastes from hot and humid areas, revealing systemic failures. Cassava showed fewer exceedances, though artisanal flours remain at risk due to practices that promote secondary contamination. In milk, AFM1 contamination is particularly alarming because exposure is indirect, driven by contaminated feed, and primarily affects vulnerable groups, including infants.

### 6.6. General Critique of Sampling Designs, Analytical Methods and Method Validation

Overall, the reviewed studies demonstrate substantial efforts to document mycotoxin contamination in the region, with a growing recognition of the intrinsic heterogeneity of mycotoxins. Accordingly, several studies incorporate multi-point sampling within lots (top, middle, and bottom of bags or containers) [54,83] and rely on composite samples to better capture the spatial variability [58]. The most elaborate investigations cover multiple stages of the food value chain, allowing for the identification of major critical points [51,103]. Some studies also account for agroecological gradients, contrasting warm and humid lowland areas with cooler highland zones, which strengthens the interpretation of dominant Aspergillus/aflatoxin versus Fusarium/fumonisin and DON profiles [53,59]. In addition, a limited but methodologically robust number of studies adopt an exposure-oriented approach by analyzing foods actually consumed by infants or young children, combined with dietary recalls and body weight data, thereby providing high internal validity for risk assessment [61,98].

Despite these advances, several structural limitations affect the overall scientific robustness. Representativeness often remains weak, as many studies rely on sampling from urban markets or easily accessible sites, which are poorly extrapolable to rural areas where production and storage predominantly occur [57,81]. Although the term “random sampling” is frequently used, it is rarely supported by a clear description of the sampling frame or probabilistic selection procedures, effectively resulting in convenience sampling. The excessive use of composite samples, while reducing analytical costs, often masks high contamination events. This prevents the reliable estimation of the true proportion of contaminated lots, limits the assessment of high exposure scenarios and associated uncertainty, and substantially reduces the ability to identify risk factors at the household or lot level [79,83]. Furthermore, most studies are cross-sectional and conducted during a single season, whereas mycotoxin contamination is strongly influenced by seasonality, storage duration, insect pressure, and interannual climatic variability. The few longitudinal studies incorporating multiple time points between harvest and prolonged storage are clearly superior for inferring causal dynamics [64]. Finally, some surveys exhibit unbalanced stratification, with large overall sample sizes but very small subgroups (e.g., warehouses or processors), while essential post-harvest metadata (actual moisture content, storage duration and conditions, packaging type, infestation, traceability) are often incomplete [61,77].

From an analytical perspective, LC–MS/MS-based approaches (or UPLC-MS/MS/TOF-MS), which remain rarely used in the region [52,68,89], are the most specific and sensitive. They enable co-occurrence analysis, provide low limits of detection and quantification, allow for comprehensive validation, and ensure good international comparability [129]. LC-FLD methods coupled with immunoaffinity columns also represent a robust reference for aflatoxin analysis when validation is adequately reported [76]. In contrast, rapid tests, ELISA, and lateral flow assays [130], widely used in the region [60,77], are useful for large-scale surveillance in resource-limited settings, but their scientific reliability remains limited in the absence of matrix-specific validation, adequate quality control, and confirmation by instrumental methods. Issues related to cross-reactivity, matrix effects, and inter-laboratory comparability are recurrent, while reporting of validation parameters is sometimes incomplete. These analytical inconsistencies further complicate the direct comparison of results across studies.

Nevertheless, some studies conducted across multiple countries using harmonized analytical procedures (e.g., Probst et al. [55], Udomkun et al. [54], and Sulyok et al. [89]) provide particularly robust datasets and enable meaningful inter-country comparisons. These studies are especially valuable from a methodological perspective, as they reduce the variability related to analytical techniques and improve data comparability. A harmonized visual synthesis of these cross-country datasets is provided in Supplementary Figures S2–S4. Notably, the reprocessed data highlight clear cross-country differences in mycotoxin profiles (Figures S2–S4). In maize, higher levels of AFT, fumonisins, and DON are observed in Uganda and the DRC compared with Tanzania and Zambia, where fumonisins predominate, while Rwanda exhibits more moderate contamination (Figure S2) [55]. In peanuts and maize flours, aflatoxin levels appear consistently higher in the DRC than in Burundi (Figure S3) [54]. In cassava flour, distinct toxin profiles emerge between countries, with BEA (~90%) and ZEN predominating in Rwanda, whereas AME, AOH, and ENN B are more prevalent in Tanzania (Figure S4) [89]. These visualizations illustrate how harmonized analytical datasets can support more robust and interpretable cross-country comparisons, in contrast to the heterogeneity observed in many other studies. While these figures primarily reflect contamination patterns, they also underscore the importance of analytical consistency as a prerequisite for meaningful comparison and interpretation across studies.

### 6.7. Public Health and Socio-Economic Implications

From a public health perspective, the converging evidence indicates that chronic low-dose exposure through staple foods (maize, peanuts, sorghum, and infant porridges) coexists with recurrent acute episodes [102]. Biomarkers, such as AFB-alb adducts, AFM1 in milk, and urinary metabolites, confirm population-level exposure and should be more widely assessed, particularly among infants, pregnant and lactating women, and in areas with high hepatitis B prevalence due to the synergistic risk for hepatotoxicity [131,132]. Economically, post-harvest losses, export rejections, and the burden on public health systems would be considerably reduced if proven practices regarding hermetic storage and improved drying, sorting, biocontrol, and control of animal feed were consistently adopted [9,130]. Across the region, three priorities emerge:

➢ Adapt strategies to agroecological gradients: Harvest calendars, drying windows, moisture thresholds, and technical advisories must be tailored to specific zones (equatorial, wet/dry savannas, highlands, and coastal areas), as contamination patterns are strongly climate dependent (Section 6.2.1).
➢ Address post-harvest and livestock vulnerabilities: Expand hermetic storage, improve warehouse aeration, adopt mechanical/optical sorting where feasible, and systematically monitor feed ingredients to prevent AFM1 transfer into milk, particularly in high-risk areas.
➢ Institutionalize integrated, risk-proportionate surveillance: Seasonal sampling plans, multi-mycotoxin LC-MS/MS methods to capture co-occurrence and emerging toxins, harmonized cross-border standards, and expanded use of rapid screening tools for large-scale sorting and early detection.

Combining agroecological targeting with improved post-harvest and feed management, alongside harmonized risk-based surveillance, offers the most realistic path toward a rapid reduction in exposure while supporting sustainable cereal and legume value chains across the region.

### 6.8. Regulatory Frameworks and Surveillance

Only Tanzania and Uganda have established national regulations, developed respectively by the Tanzania Bureau of Standards (TBS) and the Uganda National Bureau of Standards (UNBS), which set maximum limits for AFB1 (5 µg/kg) and total aflatoxins (10 µg/kg) in peanuts and maize (Table S5) [11]. Rwanda, through the Rwanda Standards Board (RSB), applies standards harmonized with those of the EAC and the Codex Alimentarius [121]. To clarify the scope and applicability of regulatory limits across the region, Table S5 provides a comparative overview distinguishing between countries with formally established national regulations and those relying on regional or international reference values (e.g., Codex or EU limits) without formal legal enforcement.

Nevertheless, surveillance remains uneven across the region. Uganda and Tanzania implement routine inspections despite limited resources [103], whereas Zambia, the DRC, and Burundi rely primarily on sporadic investigations. In the DRC, for example, monitoring activities are restricted to Kinshasa, Lubumbashi, and the Kivu region, with no national coordination. The absence of a harmonized regulatory framework and an effective surveillance system heighten the health, economic, and food security risks associated with mycotoxins. Without stringent standards, populations remain exposed to contaminated foods, contributing to liver cancer, stunting, and immunosuppression [33]. This regulatory gap undermines competitiveness, limits access to international markets [6], discourages the adoption of good agricultural practices, and delays the implementation of effective mitigation strategies, ultimately jeopardizing food safety and consumer protection. In addition, the lack of regulations for emerging mycotoxins (e.g., BEA, ENN B), despite their documented occurrence across multiple commodities (Section 4) and frequent co-occurrence with regulated mycotoxins, further increases the toxicological uncertainty. Their combined presence with aflatoxins or fumonisins may lead to additive or synergistic effects [133], which are currently not accounted for in the existing regulatory frameworks.

### 6.9. Control and Mitigation Strategies

Given the negative impact of mycotoxins on health, income and trade, developed countries worldwide have implemented robust mitigation strategies [11]. In contrast, very few African countries have established comprehensive mycotoxin policies, and where such strategies exist, their implementation remains a significant challenge. However, the African Union Commission hosts the PACA, which aims to protect crops, livestock and populations from the harmful effects of aflatoxins. The initiative seeks to enhance food security, improve public health and promote trade across the continent [109]. Unlike the DRC, where there are no formal mycotoxin control strategies in place, countries such as Tanzania and Uganda actively benefit from the PACA’s support. The DRC could consider joining this partnership to strengthen aflatoxin control measure across the food value chain [51]. Substantial evidence highlights the widespread presence of mycotoxins at hazardous levels in the staple foods consumed in the DRC, including maize, cassava, peanuts and derived products and by-products [50,51,52,54,55,56,74,75]. This alarming situation underscores the urgent need for the DRC government to develop and implement a national mycotoxin control policy. Such a policy should focus on stakeholder awareness, promoting good agricultural practices, establishing mycotoxin monitoring systems and ensuring the proper management of contaminated food products.

#### 6.9.1. Awareness

The lack of awareness among stakeholders represents a significant barrier to the effective implementation and enforcement of mycotoxin mitigation strategies [103]. It is essential that all stakeholders are well informed about the health risks mycotoxins pose to both humans and animals, as well as the regulatory standards governing their presence in food. Agricultural operators, processors and traders must be educated on the importance of adopting good agricultural practices before, during and after harvest, as well as good manufacturing, conservation and handling practices. Additionally, policy makers, including members of parliament, government officials and local authorities, should understand the critical need to pass legislation focused on mycotoxin control. Integrating mycotoxin control and management into national development plans across health, agriculture and environment sectors is imperative. Moreover, subsidies for agricultural inputs and equipment (e.g., drying, storage, monitoring and research) and the allocation of dedicated budgets are necessary to ensure the successful operation of mitigation strategies. Health personnel, particularly nutritionists, should be equipped to recommend alternatives to high-risk foods. Donor agencies should be mobilized to provide financial support to governmental mycotoxin control and monitoring initiatives. Examples of awareness campaigns have been documented in various countries. For instance, in Kenya, well-coordinated communication strategies and the active involvement of stakeholders led to the sensitization of 1000 extension workers, over 7000 farmers and 300 policy makers on mycotoxin-related issues in 2014 and 2017 [109]. In both Tanzania and Uganda, the Aflasafe initiative has been pivotal in supporting the PACA’s efforts to develop and implement awareness-raising strategies [115,134,135].

#### 6.9.2. Agricultural Practices

##### Pre-Harvest Practices

Controlling the initial contamination of crops is crucial, as the presence of pathogens after harvest and during storage largely depends on the field inoculum [135,136]. To address this issue, agricultural policies should prioritize research focused on developing improved seed varieties that are more resistant to mycotoxin-producing fungi, while considering ecological and agricultural requirements [137]. For example, researchers have identified aflatoxin-resistant varieties based on their unique phenotypic traits from 25 peanut samples collected in Kenya, Uganda and Rwanda [138]. One such variety, Serenut 2, demonstrates strong resistance to *A. flavus* and the subsequent production of aflatoxins [135]. In the DRC, the National Institute for Agronomic Studies and Research (NIASR) plays a key role in this area. One of its primary objectives is to generate, develop and adapt high-performing genetic materials for the benefit of farmers and breeders. These improved materials are designed to be resilient against major diseases and pests, contributing to mycotoxin mitigation efforts [139].

Another strategy, biological control, relies on competitive exclusion, where an atoxigenic strain competes with a mycotoxin-producing strain for the same substrate. Based on this principle, a biocontrol technology was developed by the Agricultural Research Service of the United States Department of Agriculture (USDA-ARS) [109]. This method involves inoculating crops with an atoxigenic isolate of *A. flavus* (10 kg/ha) two to three weeks before flowering. The atoxigenic strain competes with and suppresses potential aflatoxin-producing strains, significantly reducing or even eliminating aflatoxin production [109,121,135]. For over three decades, several products based on atoxigenic isolates, such as AF36 and Afla-Guard, have been approved in the USA and are used on various crops, including maize, peanuts, and cotton [109]. In Africa, the International Institute of Tropical Agriculture (IITA), in collaboration with the USDA-ARS, has developed region-specific biological control products known as AflasafeTM. These products have demonstrated up to 100% effectiveness for controlling aflatoxin contamination [103,121]. Tanzania approved AflasafeTM products in 2018, which have been marketed as Aflasafe TZ01 and TZ02 [103]. In partnership with the PACA, other countries, such as Kenya, Zambia, Malawi, Mozambique, Nigeria, Senegal, Gambia, Burkina Faso and Ghana, have integrated AflasafeTM into their aflatoxin mitigation strategy [109,115,121]. However, to maintain the effectiveness of biocontrol methods throughout the supply chain, they must be combined with good agricultural practices [140].

These are essential to reduce crop stress and enhance plant vigor, thereby minimizing susceptibility to mycotoxin-producing fungi. Key practices include effective weed management, pest and insect control, crop rotation, the use of adequate fertilizers and moisture control [9,103,121,141]. For these strategies to be effective, it is crucial to ensure that agricultural inputs, often inaccessible to smallholder farmers, are readily available.

##### Practices During and After Harvest

Poor harvesting and post-harvest handling practices significantly contribute to increased aflatoxin contamination throughout the supply chain [51,142]. Timely harvesting, immediately after crop ripening, is recommended to prevent contamination [109,135]. For instance, in Uganda, aflatoxin contamination was found to be seven times higher in late-harvested crops [103]. Simple and cost-effective methods, such as visual sorting, hulling, winnowing, grinding, washing, soaking and roasting, can substantially reduce mycotoxin levels in grains [135].

Efficient drying practices are critical, aiming to quickly reduce the moisture content to between 10 and 12%. Traditional solar drying, commonly used by smallholder farmers, often falls short due to variability in sunlight and alternation of day and night, making it challenging to achieve the required moisture levels consistently [121]. To address this limitation, government support could facilitate access to advanced drying technologies, such as electric or hybrid dryers that combine solar energy with biomass [9]. Proper storage is equally important. Crops should be kept in well-ventilated structures that maintain temperatures below 18 °C and relative humidity between 12 and 13% [109,135]. Industrial operations typically utilize silos equipped with electronic sensors and moisture meters, while smallholder farmers could adopt metal silos and airtight bags to preserve grain quality effectively.

#### 6.9.3. Mycotoxin Monitoring Throughout the Value Chain

Mycotoxin monitoring in the study area is very weak in the DRC [52], Rwanda [143], and Burundi [143], but comparatively more advanced in Uganda [135,143] and Tanzania [143,144]. Indeed, Uganda has completed several national studies as well as established standards for maize and groundnuts [11]. It has adopted Aflasafe UG for biocontrol [140] and promoted solar drying [145] and the use of hermetic PICS bags [146]. Tanzania has more structured national programs [144] and standards for maize and groundnuts [11]. It has adopted Aflasafe TZ [140], community dryers, and the use of PICS bags [146]. The DRC has neither a coordinated national surveillance system nor specific standards, although very limited academic research studies are available [52]. Nevertheless, Aflasafe RDC 01 has been introduced in the country [140,147]. In Rwanda, no national monitoring program currently exists. A few ad hoc studies have been conducted [70], notably with the support of the FAO through projects focused on improved storage and the use of hermetic bags [148]. Finally, in Burundi, mycotoxin surveillance is virtually non-existent, as the country has neither specific regulatory frameworks nor monitoring programs. The actual extent of food contamination by mycotoxins remains largely under-documented [71].

#### 6.9.4. Management of Contaminated Foods and Feeds

Completely eradicating mycotoxins from food products is nearly impossible due to the unpredictable environmental factors that influence contamination. However, several techniques, ranging from the simple sorting of moldy foods to more complex biological and non-biological methods such as ammonification, ozonization and photosensitization, have been studied to reduce mycotoxin levels [149]. For example, dehulling of maize used in the preparation of meals, such as porridge, has been shown to reduce aflatoxin contamination by more than 50% [150]. In addition to sorting, household-level practices, such as the washing and heating of grains, can further reduce mycotoxin contamination in food. Another major challenge in controlling mycotoxin contamination is the reintroduction of contaminated products into the supply chain, either for human consumption or as ingredients in animal feed formulations [6,109]. This contributes to a loss of income for producers. Providing financial compensation to producers who suffer losses due to contaminated crops can discourage the illegal reintroduction of such products into the market. As an alternative use, contaminated food could be repurposed as biomass for industrial processes.

### 6.10. Limitations of the Review

This review is based on publicly available data, and some non-indexed or unpublished local studies may not have been captured. Additionally, the variability in analytical methods, limits of detection, and reporting units limits full comparability across studies. Differences in sampling design, representativeness, and study periods further contribute to heterogeneity in the reported data. Nevertheless, this synthesis provides a representative overview of the current state of mycotoxin contamination in foodstuffs across the region, highlighting the consistent patterns, key hotspots, and major drivers of exposure.

## 7. Conclusions and Future Directions

This review highlights that mycotoxin contamination remains an important and persistent problem in the DRC and neighboring countries. It is largely driven by climatic conditions and agroecological gradients. Warm and humid climates favor the proliferation of Aspergillus species and the production of aflatoxins, whereas cooler and wetter highland areas are conducive to the development of Fusarium species and contamination by fumonisins and DON. These dynamics occur within a closely interconnected plant–food–animal–human ecosystem, illustrated in particular by the presence of AFM1 in milk, confirming the continuity of exposure pathways along food supply chains.

The data generated over the past fifteen years consistently confirm the frequent contamination of foods and animal feeds, with the progressive accumulation of mycotoxins along supply chains exacerbated by inadequate post-harvest practices. Their co-occurrence, including emerging mycotoxins, increases health risks, particularly among vulnerable populations such as young children, and also results in significant socio-economic consequences. Nevertheless, regional assessment of the problem remains constrained by major gaps, including fragmented and heterogeneous datasets across countries, insufficient documentation of emerging mycotoxins, a scarcity of biomarker-based multi-exposure studies, and a lack of cost-effectiveness analyses of mitigation strategies, especially in large areas of the DRC and in other poorly documented countries.

Cross-border trade, largely informal, represents an additional pathway for mycotoxin dissemination, amplified by non-harmonized standards and weak border control systems. As highlighted by the spatial patterns of contamination and connectivity described in this review (Figure 1), these dynamics reinforce the regional nature of the problem and the need for coordinated responses. In response to these challenges, an integrated and coordinated approach involving researchers, policymakers, public institutions, donors, value chain actors, and local communities is essential.

Future priorities include: (i) harmonized surveillance based on multi-mycotoxin analytical methods; (ii) the development of longitudinal exposure studies, particularly mother–child cohorts; (iii) improved characterization of cross-border trade flows; (iv) identification of mycotoxin-resistant crop varieties; (v) development of contamination maps; and (vi) evaluation of mitigation strategies adapted to local realities. In this context, the establishment of a multidisciplinary Centre of Excellence in the DRC, supported by strengthened cooperation among regional organizations, represents a key strategic lever for achieving a sustainable reduction in mycotoxin exposure in Central and Eastern Africa.

## Data Availability

No new data were created or analyzed in this study.

## Supplementary Materials

The following supporting information can be downloaded at: https://www.mdpi.com/article/10.3390/toxins18040182/s1, Table S1. Summary of reported occurrence of mycotoxins in maize and maize-derived products in the Democratic Republic of the Congo and neighboring countries (2009–2024) [50,51,52,53,54,55,56,57,58,59,60,61,62,63,64,65,66,67,68,69,70,71,151,152]; Table S2. Summary of reported occurrence of mycotoxins in peanut and peanut-derived products in the Democratic Republic of the Congo and neighboring countries (2009–2024) [50,52,54,59,60,61,62,67,68,74,75,76,77,78,79,80,81,82,83,84,85,151,152,153]; Table S3. Summary of reported occurrence of mycotoxins in cassava and cassava-derived products in the Democratic Republic of the Congo and neigh-boring countries (2009–2024). [52,53,54,68,78,87,88,89,151,152]; Table S4. Summary of reported occurrence of mycotoxins in selected food commodities other than maize, peanuts, and cassava (including sorghum, soybean, beans, millet, milk, insects, fruits, sunflower, feed, and feed ingredients) in the Democratic Republic of the Congo and neighboring countries (2009–2024) [50,54,60,61,78,90,91,92,93,94,95,96,151,152,153]; Table S5. Overview of national, regional (EAC), and international (Codex/EU) maximum limits for selected mycotoxins in maize, peanuts, and milk in the Democratic Republic of the Congo and neighboring countries. Figure S1. Administrative map of the Democratic Republic of Congo (DRC) showing provincial boundaries and the nine neighboring countries referenced in Section 3.1. Figure S2: Comparative visualization of reported levels of AFT, fumonisins, and DON in maize across selected countries (DRC, Rwanda, Tanzania, Uganda, and Zambia), based on data extracted from Probst et al. [55]. Figure S3: Comparative visualization of AFT levels in selected food commodities (beans, cassava, maize, milk, peanuts, sorghum, and soybean) between the DRC and Burundi, based on data extracted from Udomkun et al. [54]. Figure S4: Comparative visualization of the occurrence (%) of multiple mycotoxins (AFB1, AME, AOH, BEA, DON, ENN B, FB1, FB2, NIV, OTA, and ZEN) in selected cassava flour samples from Tanzania and Rwanda, based on data extracted from Sulyok et al. [89].
