# Supplementary material for: A Critical Review of Mycotoxin Contamination in Food and Feed in the Democratic Republic of the Congo and Neighboring Countries: Challenges and Future Directions
Source: Toxins (Basel). 2026 Apr 10;18(4):182. doi: 10.3390/toxins18040182 (PMC13119991; doi:10.3390/toxins18040182)
Supplement: Supplementary file 1 [file toxins-18-00182-s001.zip › toxins-3919899-supplementary.pdf]

toxins

MDPI

Table S1. Summary of reported occurrence of mycotoxins in maize and maize-derived products in the Democratic Republic of the Congo and neighboring countries (2009–2024). The table compiles data from the reviewed studies, including food matrices, sampling sources, mycotoxin types, number of samples, prevalence (% positive samples), concentration levels (mean and range, µg/kg), proportion of samples exceeding regulatory limits (%), analytical methods, and references.

**Countries**	**Food matrix**	**Sampling source**	**Mycotoxin**	**Number of samples**	**Positive samples (%)**	**Mean (µg/kg)**	**Range (µg/kg)**	**non-compliant samples (%)**	**Methods LOD**	**References**
**DRC**	Maize flour	Markets (Western)	AFB1	8	87.5	2.2	1.2-5.9	25[1]	UPLC-MS/MS	[52]
			AFT		100	3.6	1-7.7	25[1]		
			FB1		87.5	14.5	1.8-39.2			
			DON		75	4.5	2.3-7.3			
			NIV		75	13.9	12.3-16.1			
			BEA		12.5	20.6	20.6			
			ENN B		25	7.6	7.6			
			AOH		12.5	4.5	4.5			
			AME		12.5	19.9	19.9			
	Freshly harvested dry maize	value chain (South Eastern)	AFT	64	100	3.2	0.3-18.5	10[2]	Reveal Q+	[56]
	Stored maize flour			38	100	148.9	2.05-905.1	100[2]		

	Maize flour	Markets (Eastern)	AFT	9	100	47.9	2.5-320	77.5[1]	Reveal Q+		[54]
	Maize flour	House-hold (Eastern)	AFT	50	100	32.5	2.5-325	88[1]	Reveal Q+		[53]
	Maize grain	Farmers (West-ern)	AFT	50	32	20.64	3.1-103.89	22[2]	LC-FLD	0.31 - 0.69	[51]
		Markets (West-ern)		30	100	560.485	3.61-2806.5				
	Maize seed	Markets (Eastern)	AFT	12		63	393		ELISA		[55]
		Markets (Easter)	Fumonisi ns	12		2000	9000				
		Market (Eastern)	DON	12		1000	4000				
	Maize grain	Markets (South Eastern)	AFT	40	95	257.5	3.1-439.2		TLC, LC-FLD		[50]
			OTA	40	45	61.9	0.5-164.2				
			Fumonisi ns	40	100	2207.5	17.5-6258.2				
			ZEN	40	92.5	247.8	24-811.2				
**ZAMBIA**	Maize stored	House-holds (South-ern, Cen-tral,	Aflatox-ins		21.4		0.7-108.39		ELISA		[57]

		Northern)									
			Fumonisins		96.4		20- 21440				
	Maize ear	Farmers (Southern and Central)	Aflatoxins	114	100	5.4	0.2-10		ELISA		[58]
			Fumonisins	114	100	73300	3700-192000				
	Maize seed	Markets	AFT	28		7	108	9.1[2]	ELISA		[55]
			Fumonisins	28		2000	21000				
	Maize grain	Agricultural warehouses and markets (Southern)	AFT	43		12		20[2]	Reveal Q+	2	[59]
		agricultural warehouses and markets (Northern)		34		25		9[2]			

**UGANDA**	Maize seed	Markets	AFT	17		95	435	20 (>100 µg/Kg)	ELISA		[55]
			Fumonisins	17		2000	19000				
			DON	17		800	8000				
	Maize grain	House-hold (North-ern)	AFT	116	35	6.77	0.00-99.96	26[1]	ELIZA	3	[60]
			Fumonisins	125	67	3.49	0.00-43.60	42[1]		200	
			OTA	113	12.	0.37	0.00-5.97	1[1]		1.9	
			DON	97	77	0.9279	0.00-8.175	25[1]		200	
	Maize flour	House-hold (South Western)	AFT	31	74.2	34.1	Nd-336.5	58[2]	ELISA		[61]
	Maize	Value chain category (West-ern)	AFT	40	45	0.045			ELISA		[62]
**TANZA-NIA**											
	Maize	House-holds (Eastern, Northern	AFB1	60	50	65	3-1081	28[2]	UPLC-TOFMS	0.5-8	[63]

	and South Western)									
		FB1		73	1361	16-18184			25-400	
		FB2		48	2921	178-38217			25-400	
		DON		63	490	68-2196	5[1]		25-400	
Maize seed	Market	AFT	5		2			ELISA		[55]
		Fumonosins	5		1000					
Maize porridge	House-holds (South-ern)	FB1/FB2	34	100				UPLC-MS/MS	0.2	[64]
	House-holds (North-ern)	FB1/FB2	34	100						
	House-holds (Central)	FB1/FB2	33	100						
	House-holds (Central) (six months after har-vest)	AFB1	33	100	10.21				0.05	

		Households (Central) (six months after harvest)	AFT	33	100	14.91					
	Maize flour	Households (Northern)	AFT		32		0.11-386	5[2]	LC-FLD	0.01	[65]
			DON		44		57-825	2[2]			
			Fumonisins		83		63-2284	12[2]		47	
	Maize flour for infant	Households (Northern)	AFT	67	58		0.33-69.47	23[2]	LC-FLD	0.01	[66]
			Fumonisins	67	31		48.4-1224.6	48[1]		47	
	Maize grain	Rural households	AFT	200	49.5	12.48		11[1]	LC-FLD	0.07	[67]
**RWANDA**											
	Maize seed	Markets	AFT	16			0.3		ELISA		[55]
			Fumonisins	16		300	1000				
			DON	16			500				

	Maize flour	Market	AFB1	27	89	2.3	0.3-11.1		LC-MS/MS		[68]
			AFT	24	100	4.03	0.8-16.8				
			OTA	27	11	3.7	Nd-3.7				
			FB1	27	67						
	Maize	Value chain	AFT	227	100	6.69	0 -100.9	10[2]	Reveal Q+	2	[69]
			Fumonisins		100	0.15	0 -2.3				
	Maize grain	Farmers (Volcanic region)	AFT	60		40.45			ELISA, UHPLC-MS/MS		[70]
		Farmers Eastern Savannah)	AFT	60		4.66					
**BURUNDI**											
	Maize grain	Markets	AFT	10	100	38.7	2.7-330	60[1]	Reveal Q+		[54]
	Maize flour		AFT	10	100	41.9	3.2-350	70[1]			
	Maize		AFT	380	79			3[2]	Reveal Q+	2-150	[71]

[1]EU (European Union) maximum limits [151]: 2 μg/kg for AFB1 (aflatoxin B1) and 4 μg/kg for total aflatoxins, [2]CAC (Codex Alimentarius Commission) maximum limits [152]: 5 μg/kg for AFB1(aflatoxin B1) and 10 μg/kg for total aflatoxins.

Table S2. Summary of reported occurrence of mycotoxins in peanut and peanut-derived products in the Democratic Republic of the Congo and neighboring countries (2009–2024). The table compiles data from the reviewed studies, including food matrices, sampling sources, mycotoxin types, number of samples, prevalence (% positive samples), concentration levels (mean and range, µg/kg), proportion of samples exceeding regulatory limits (%), analytical methods, and references.

Countries	Food matrix	Sampling source	Mycotoxin	Number of samples	Positive samples (%)	Mean (µg/kg)	Range (µg/kg)	non-compliant samples (%)	Methods	LOD	References
**DRC**											
	Peanut paste	Markets (Western)	AFB1	5	80	1.3	01-2.3	20[1]	UPLC-MS/MS		[52]
	Peanut paste		AFT	5	80	2.4	0.1-6.6	20[1]			
			BEA	5	20	225.4	225.4				
	Peanut flour	Market (Eastern)	AFT	2	100	1027.5	470-1620	0	Reveal Q+		[54]
	Raw peanut	Markets (Western)	AFT	20	100	206.20	2.19-1258		LC-FLD		[75]
			AFB1	20	100	97.39	2.19-544	75[2]			
	Peanut	Markets (South Eastern)	AFB1	40	100	368.7	2.5-574.2		TLC and LC-FLD		[50]
	Peanut		OTA	40	72.5	11.6	1.4-111.5				
	Raw peanuts	Markets	AFB1	60	72	229.07	1.5-937	70[2]	TLC		[74]

		(Western)									
**ZAMBIA**											
	Peanut	Agricultural warehouses and markets (Southern)	AFT	43		22	5.1-40.5	100[1]	Reveal Q+	2	[59]
		Agricultural warehouses and markets (Central)		222		90	3.5-361	51[1]			
	Peanuts kernels	Markets	AFB1	163			Nd-11100		ELISA	1	[77]
	milled peanut powder			39			1-3000				
	Raw peanuts	Markets (Central)	AFT	92	55.4	0.43	0.014-48.6	6.5[2] 12[1]	LC-FLD	0.10	[76]
			AFB1	92	44.6	0.45	0.015-46.6			0.05	
**UGANDA**											

	Peanuts	Value chain category (District of Soroti)	AFT	40	30	0.052			ELISA		[62]
	Peanut flour	South West region	AFT	11	100	96.5	6.2-297.3	91[2]	ELIZA		[61]
	Peanut Flour	Households of Kampala	AFT	93	76.3	37.9	0.04 - 296.4		ELIZA	0.04	[84]
	Peanut	Value chain (Northern)	AFT	79	100		8.93-210.93	36.6[1]	Reveal Q+	2-150	[83]
		Value chain (Eastern)	AFT	54	100		2.9-383.25	50			
	Peanut stored	Farmers and household	AFT	179		12.6	0-1327	47[1]	Reveal Q+	2	[82]
	Peanut seeds	Household	AFT	66	79	6.87	0.00-56.31	71[1]	ELISA		[60]
	Peanut	Markets of Kampala	AFB1	33	63	103.1	0.00-540	34[3]	LC-MS/MS	0.8	[81]
			AFT	33	82	180.7	0.00-849	55[3]			

	Peanut seeds	Markets (Eastern)	AFT	14	28.5		1.6-516		TLC and ELISA		[80]
	Peanut flour	Markets (Northern)		18	50		6.2-11732				
	Peanut seeds	Markets	AFT	5			940	40[3]	VICAM AflaTest fluorometric		[79]
	Peanut paste		AFT	5	100		725	100[3]			
	Peanuts	Household (South Western)	AFT	3	100	11.5	0.00-55	100[1]	Vicam aflatest		[78]
**TANZANIA**											
	Peanut	Rural households	AFT	180	96.1	6.37	0.04-40.31	30.6[1] 17[2]	LC-FLD	0.07	[67]
	Peanut		AFB1	180	74.4	5.02	0.26-38.30			0.6	
	Peanut-enriched flour	Manufacturers	AFB1	65	100		1.24-60.64	71[5]	LC-FLD	0.53	[85]
			AFT	65	100		2.83-92.4	71[5]			
**RWANDA**											

	Peanut flours	Markets	AFB1	27	100	27.66	5.5- 66.8		LC-MS/MS		[68]
			AFT	23	100	52.27	9.2-126.6				
			FB1	27	11	16.3	Nd-16.3				
			OTA	27	33	2.8	Nd-2.8				
**BU-RUNDI**											
	Peanut flour	Markets	AFT	10	100	824	310-2410	100[1]	Reveal Q+		[54]
	Peanuts		AFT	120	82			6[4]	Reveal Q+	2-150	[82]

[1]EU (European Union) maximum limits [151]: 2 µg/kg for AFB1 (aflatoxin B1) and 4 µg/kg for total aflatoxins, [2]CAC (Codex Alimentarius Commission) maximum limits [152]: 5 µg/kg for AFB1(aflatoxin B1) and 10 µg/kg for total aflatoxins, [3]US FDA (US Food and Drugs Administration) maximum limits [153]: 20 µg/kg for total aflatoxins

Table S3. Summary of reported occurrence of mycotoxins in cassava and cassava-derived products in the Democratic Republic of the Congo and neighboring countries (2009–2024). The table compiles data from the reviewed studies, including food matrices, sampling sources, mycotoxin types, number of samples, prevalence (% positive samples), concentration levels (mean and range, µg/kg), proportion of samples exceeding regulatory limits (%), analytical methods, and references.

Countries	Food matrix	Sampling source	Mycotoxin	Number of samples	Positive samples (%)	Mean(µg/kg)	Range (µg/kg)	non-compliant samples (%)	Methods	LOD	References
**DRC**											
	Dried root	Household (Eastern)	AFT	30	100	3.5	2.6-5	20[1]	Reveal Q+		[53]
	Casava flour	Market (Western)	AB1	5	40	0.2	0.1-0.3	0[1]	LC-MS/MS		[52]
			AFT	5	40	0.35	0.1-0.6	0[1]			
			FB1	5	80	8.9	2.1-16.4				
			DON	5	60	1	0.1-2				
			BEA	5	20	2.6	2.6				
			ENN B	5	20	0.2	0.2				
**ZAMBIA**	Cassava (Chips and flour)	Farmers	AFT	22			<1-16	5[2]	VICAM AflaTest ® immunoaffinity fluorometric	1	[87]
**UGANDA**											

	Cassava	House-hold (South western)	AFT	18	100	16.0		61.1[1]	Vicam aflatest		[78]
	Cassava flour	Value chai (Farmer and whole-saler)	AFB1	48	19.8	1.7	13.1-298	100[1]	LC-MS/MS	0.2-1.75	[88]
			AFT		32.2	38.9	1.76	93.5[1]			
			OTA		7.3			100[1]			
	Cassava chips	Value chain (Farmer and whole-saler)	AFB1	48	15.6	13.4	2.42-420	100[1]			
			AFT		25.1	36.1	38.9	100			
			OTA		5.2			100			
**TANZA-NIA**											
	Cassava flour	House-holds and mar-kets	AFB1	405	10.6		76.6	32.5[6]	LC-MS/MS	0.002-20	[89]
			ZEN		38.0		8493	11.68[6]			
			AME		49.9		1430				
			BEA		81.2		527				
**RWANDA**											

	Cassava flour	Market	AFB1	27	33	1.8	Nd-1.8		LC-MS/MS		[68]
			AFT	27	18.5	0.98	0.1-2.7				
			OTA	27	-	2.53	Nd-3.7				
	Cassava flour	House-holds and mar-kets	BEA	222	89.6		280		LC-MS/MS	0.002 -20	[89]
			ZEN		46.8		2826	11.8[1]			
			ENN B		30.6		50.4				
**BU-RUNDI**											
	Dried root	Market (Eastern)	AFT	8	100	3.7	2.5-5.4	61.2[1] 24[2]	Reveal Q+		[54]
	Cassava flour			10	100	2.8	1.9-4.6				

[1]EU (European Union) maximum limits [151]: 2 µg/kg for AFB1 (aflatoxin B1) and 4 µg/kg for total aflatoxins, [2]CAC (Codex Alimentarius Commission) maximum limits [152]: 5 µg/kg for AFB1(aflatoxin B1) and 10 µg/kg for total aflatoxins.

Table S4. Summary of reported occurrence of mycotoxins in selected food commodities other than maize, peanuts, and cassava (including sorghum, soybean, beans, millet, milk, insects, fruits, sunflower, feed, and feed ingredients) in the Democratic Republic of the Congo and neighboring countries (2009–2024). The table compiles data from the reviewed studies, including food matrices, sampling sources, mycotoxin types, number of samples, prevalence (% positive samples), concentration levels (mean and range, µg/kg), proportion of samples exceeding regulatory limits (%), analytical methods, and references.

Countries	Food commodities	Sampling source	Mycotoxin	Number of samples	Positive samples (%)	Mean(µg/kg)	Range (µg/kg)	non-compliant samples (%)	Methods	LOD	References
**DRC**	Sorghum grain	Markets (Eastern)	AFT	11	100	4.1	2.5-5.5	63.6[1]	Reveal Q+		[54]
	Sorghum flour			7	100	4.9	3.1-6.5	75[1]			
**UGANDA**	Sorghum flour	Household (South Western)	AFT	11	66.7	12	Nd-25.54	58.3[2]	ELISA		[61]
	Sorghum grain	Household (Northern)	AFT	125	80	10.24	0.00-62.27	77[1]	ELISA		[60]
			Fumonisins	127	93	4.4023	0.00-37.0157	71[1]			
			OTA	128	67	7.44	0.00-46.25	49[1]			
			DON	90	57	0.82	0.00-17.42	24[1]			
	Sorghum	Household	AFT	22	100	15.2	0.00-55	77.2[1]	VICAM Aflatest		[78]

	(Flour and porridge)	(South Western)								
**BURUNDI**	Sorghum grain	Markets	AFT	12	100	7.1	5.6-490	100[1]	Reveal Q+	[54]
	Sorghum flour			5	100	6.1	4-8.5	100[1]		
	Sorghum germé			3	100	6.2	5.2-6.9	100[1]		
**DRC**	Beans	Markets (Eastern)	AFT	10	100	3.5	1.9-6.4	20[1]	Reveal Q+	[54]
		Markets	AFT	30	80	215.5	1.4-365.3		TLC and LC-FLD	[50]
			Fumonisins	30	83.3	243.5	3.2-321.1			
			OTA	30	82.4	47.8	1.2-89.1			
			ZEN	30	90	185.2	12.5-273.2			
**BURUNDI**	Beans	Markets	AFT	21	100	3.9	2.5-6.6	33.3[1]	Reveal Q+	[54]
**DRC**	Soybean grain	Markets (Eastern)	AFT	3	100	3.7	2.8-4.2	33.3[1]	Reveal Q+	[54]
	Soybean flour			4	100	4.1	2.3-5.5	50[1]		

**UGANDA**	Soybean (Raw material)	Caregivers of children (Northern)	AFB1	46	12.7	0.649	0.060-1.220	46[1]	ELISA	[90]
			AFT	46	12.7	1.187	0.590-4.940	46[1]		
**RWANDA**	Soybean flour	Farmers	AFT	300	3	2.4	1-11.2	1[1]	ELISA and LC-MS/MS	[91]
**BURUNDI**	Soybean grain	Markets	AFT	8	100	3.4	2.3-4.1	12.5[1]	Reveal Q+	[54]
	Soybean flour			5	100	6.9	3.5-12.3	80[1]		
**UGANDA**	Millet flour	Household (South Western)	AFT	14	50	11.7	Nd-46.51	43[2]	ELISA	[61]
	Millet (raw material)	Caregivers of children (Northern)	AFB1	92	25.4	0.347	0.010-2.000			[90]
			AFT	92	25.4	0.709	0.080-1.350			
	Millet grain	Household	AFT	78	21	1.46	0.00-51.37	12[1]	ELISA	[60]

		(Northern)									
			Fumonisins	78	45	0.76	0.00-30.28	15[1]			
			OTA	78	42	1.32	0.00-5.53	5[1]			
			DON	65	38	0.15	0.00-0.67				
	Millet (Flour and porridge)	Household (South Western)	AFT	33	100	14.0	0.00-55	90.9[1]	Vicam Aflatest		[78]
**DRC**	Milk products	Market (Eastern)	AFM1	10	100	37.3	4.8–261.1	40[1]	ELISA		[54]
**BURUNDI**		Markets	AFM1	16	100	32.5	8.2–82.8	37.5[1]	ELISA		[54]
**ZAMBIA**	*S. rautanenii*	Markets	AFT	22		57	3.4-128.6	81[2]	Reveal Q+	2	[93]
	V. lanciflora			7		12	6.6-18.9	71.4[2]			
	Ziziphus spp.			10		6	Nd-24.4	10.0[2]			
	T. garckeana			17		11	3.9-23.2	53[2]			
**ZAMBIA**	Gynanisa		AFT	49		11	2.9-24.4	40.6[2]	Reveal Q+	2	[92]
	Gonimbrasia			44		12	3.4-25.1	54.8[2]			
	Macrotermes			4		24	16-36.8	100[2]			

**TANZANIA**	Sunflower seeds	District of Singida	AFB1	40	15		LOD-218	50[1]	RP-LC		[95]
	Unrefined sunflower oils			21	80.9		LOD-2.56	17.65[1]			
**TANZANIA**	Sunflower seeds (Round I)	District of Dodoma	AFT	7	71	59.6	1.7-280.6	80[3]	ELISA	1.4	[94]
	Sunflower cakes (Round I)	District of Dodoma		7	100	33.5	1.9-88.2	57[3]			
	Sunflower seeds (Round II)	District of Morogoro		6	50	118.6	2.8-662.7	67[3]			
	Sunflower cakes (Round II)	District of Morogoro		6	100	149	2.7-536	67[3]			
**RWANDA**	Feed and Feed Ingredients	Dairy Farmers	AFT	1180		108.83		85[1]	ELISA		[96]
		Poultry Farmers		1726		103.81					
		Feed Vendors		365		88.64					
		Feed Processors		57		94.95					
		Dairy Farmers	Fumonisins	1180		1520					
		Poultry Farmers		1726		1210					

Feed Vendors	365	1480
Feed Processors	57	1030

[1]EU (European Union) maximum limits [151]: 2 μg/kg for AFB1 (aflatoxin B1), 4 μg/kg for total aflatoxins and 0.05 μg/kg for AFM1 (aflatoxin M1), [2]CAC (Codex Alimentarius Commission) maximum limits [152]: 5 μg/kg for AFB1(aflatoxin B1) and 10 μg/kg for total aflatoxins, [3]US FDA (US Food and Drugs Administration) maximum limits [153]: 20 μg/kg for total aflatoxins

Table S5. Overview of national, regional (EAC), and international (Codex/EU) maximum limits for selected mycotoxins in maize, peanuts, and milk in the Democratic Republic of the Congo and neighboring countries.

Country/ Authority	Regulatory framework status	Commodity	AFB1 (μg/kg)	AFT (μg/kg)	AFM1 (μg/kg)	Fumonisins (μg/kg)	DON (μg/kg)	Reference/ Authority	Notes
**DRC**	No formal regulation (Codex/EU used as reference)	Maize	-	-	-	-	-	Codex/EU (non-binding)	No formal national regulation; international limits used as reference without formal enforcement
**DRC**	No formal regulation (Codex/EU used as reference)	Peanuts	-	-	-	-	-	Codex/EU (non-binding)	No formal national regulation; international limits used as reference without formal enforcement
**DRC**	No formal regulation (Codex/EU used as reference)	Milk			-			Codex/EU (non-binding)	No formal national regulation; international limits used as reference without formal enforcement
**Uganda**	National regulation established	Maize	5	10	-	-	-	UNBS	National standards formally established and enforced
**Uganda**	National regulation established	Peanuts	5	10	-	-	-	UNBS	National standards formally established and enforced
**Tanzania**	National regulation established	Maize	5	10	-	-	-	TBS	National standards formally established and enforced
**Tanzania**	National regulation established	Peanuts	5	10	-	-	-	TBS	National standards formally established and enforced

**Rwanda**	Regional standards applied (EAC/Codex)	Maize						EAC/Codex	No independent national limits; regional/international standards applied
**Rwanda**	Regional standards applied (EAC/Codex)	Peanuts	-	-	-	-	-	EAC/Codex	No independent national limits; regional/international standards applied
**Burundi**	No formal regulation (Codex/EU used as reference)	Maize	-	-	-	-	-	Codex/EU (reference values, non-binding)	No formal national regulation; international limits used as reference without formal enforcement
**Burundi**	No formal regulation (Codex/EU used as reference)	Milk			-			Codex/EU (reference values, non-binding)	No formal national regulation; international limits used as reference without formal enforcement
**Zambia**	No formal regulation (Codex/EU used as reference)	Maize	-	-	-	-	-	Codex/EU (reference values, non-binding)	No formal national regulation; international limits used as reference without formal enforcement
**Codex Alimentarius**	International benchmark	Maize	5	10	-			CAC	Non-binding international reference values used for risk management and trade
**European Union**	International regulatory standard	Maize	2	4	-	1000	750	EC	Legally binding within EU; widely used as reference standard in international trade
**European Union**	International regulatory standard	Peanuts	2	4	-	1000	750	EC	Legally binding within EU; widely used as

	reference standard in international trade

Abbreviations: CAC, Codex Alimentarius Commission; EAC, East African Community; EU, European Union; UNBS, Uganda National Bureau of Standards; TBS, Tanzania Bureau of Standards.

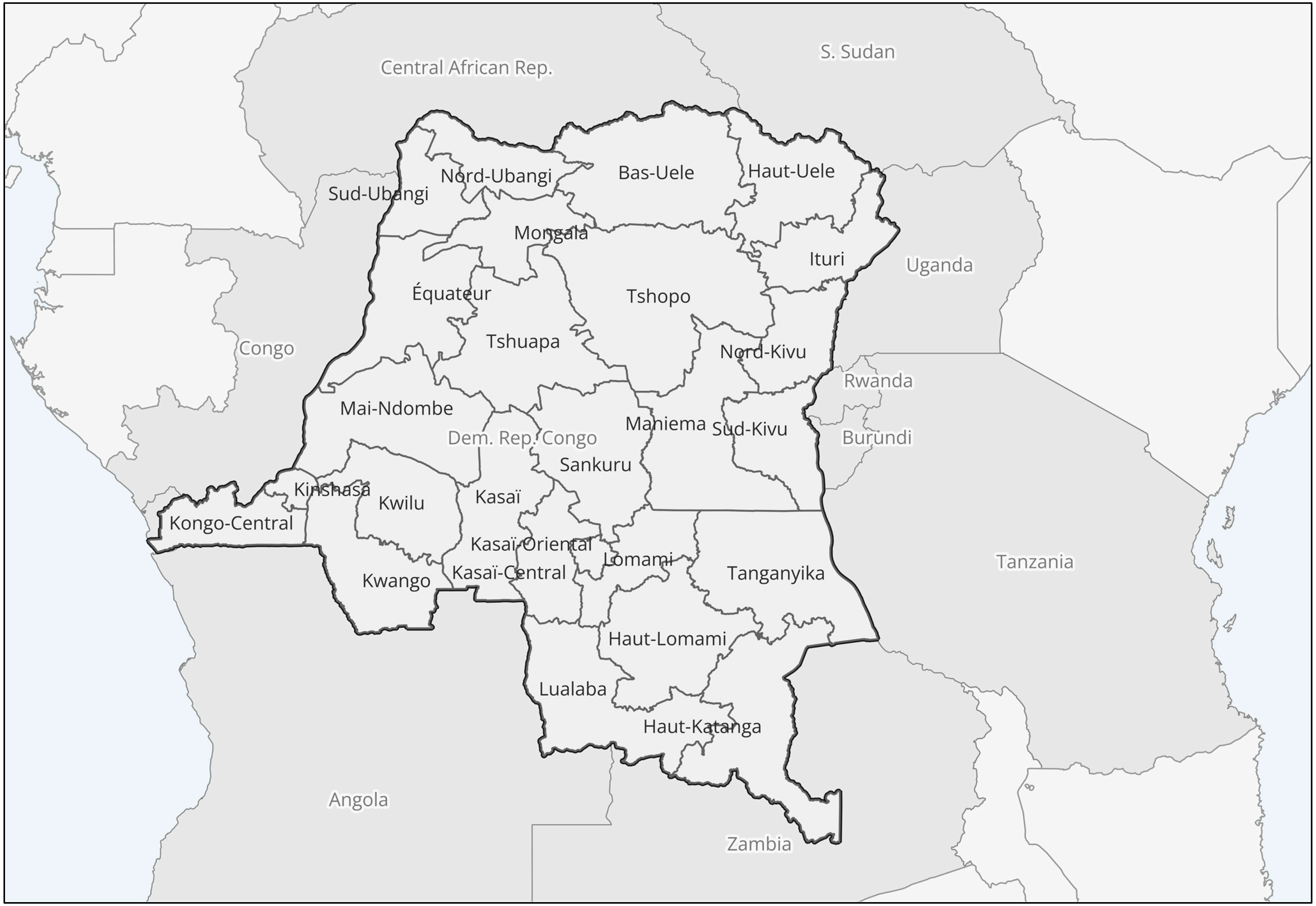
Central African Rep.
S. Sudan
Nord-Ubangi
Bas-Uele
Haut-Uele
Sud-Ubangi
Mongala
Ituri
Uganda
Équateur
Tshopo
Tshuapa
Congo
Nord-Kivu
Rwanda
Mai-Ndombe
Maniema
Sud-Kivu
Dem. Rep. Congo
Burundi
Sankuru
Kinshasa
Kwilu
Kasaï
Kongo-Central
Kasaï-Oriental
Lomami
Tanganyika
Tanzania
Kwango
Kasaï-Central
Haut-Lomami
Lualaba
Haut-Katanga
Angola
Zambia

Figure S1. Administrative map of the Democratic Republic of Congo (DRC) showing provincial boundaries and the nine neighboring countries referenced in Section 3.1. The broader African context is included for geographic orientation. DRC national and provincial boundaries (ADM0 and ADM1) were obtained from GADM, and country boundaries from Natural Earth; all layers were processed and rendered by the authors using QGIS.

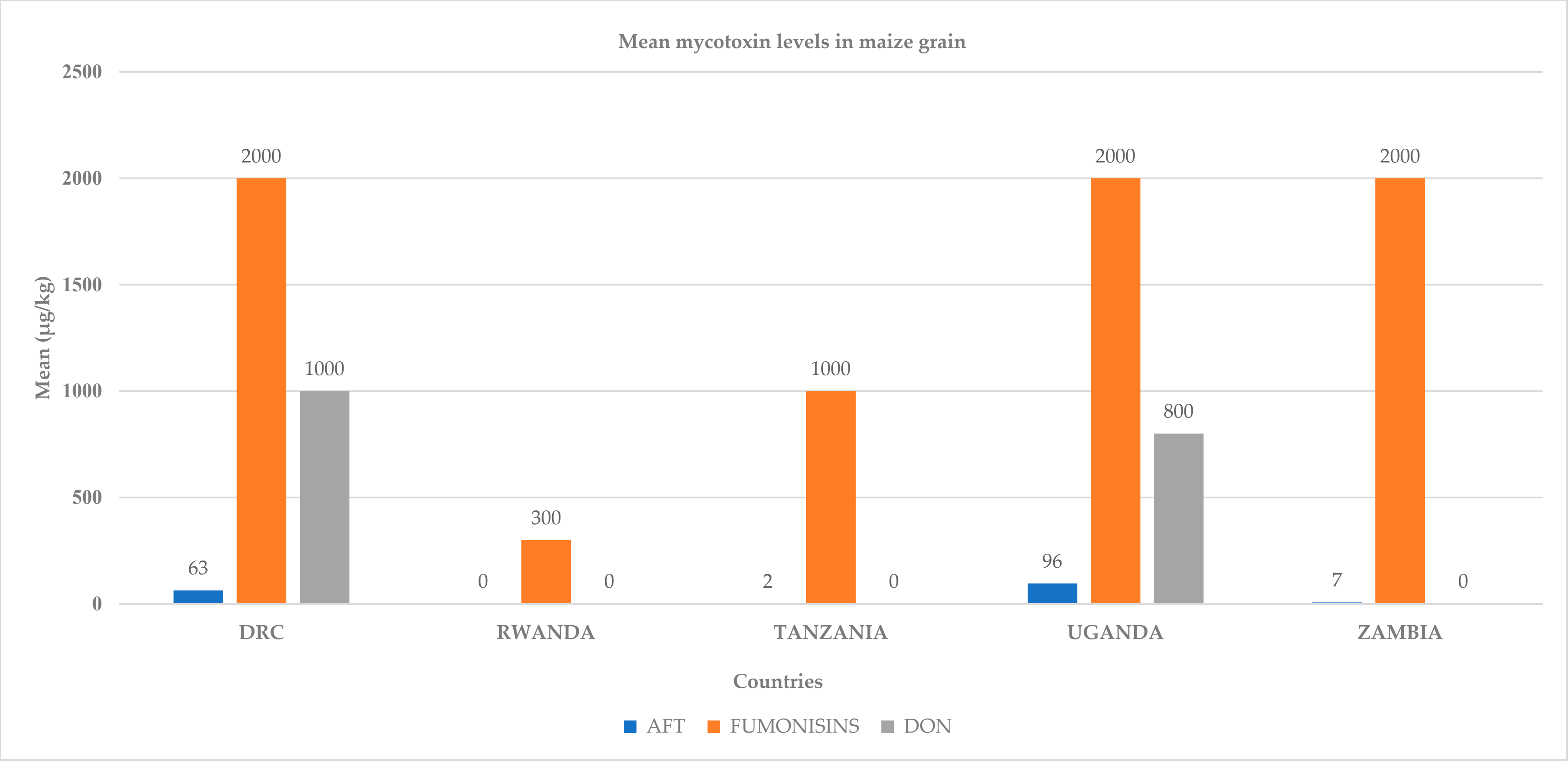

Figure S2. Comparative visualization of reported levels of AFT, fumonisins, and DON in maize across selected countries (DRC, Rwanda, Tanzania, Uganda, and Zambia), based on data extracted from Probst et al. [55]. Values represent reprocessed quantitative data from the original study, harmonized to enable cross-country comparison. The figure illustrates differences in dominant mycotoxin profiles, with higher combined contamination observed in Uganda and the DRC, while fumonisins predominate in Tanzania and Zambia and more moderate levels are observed in Rwanda.

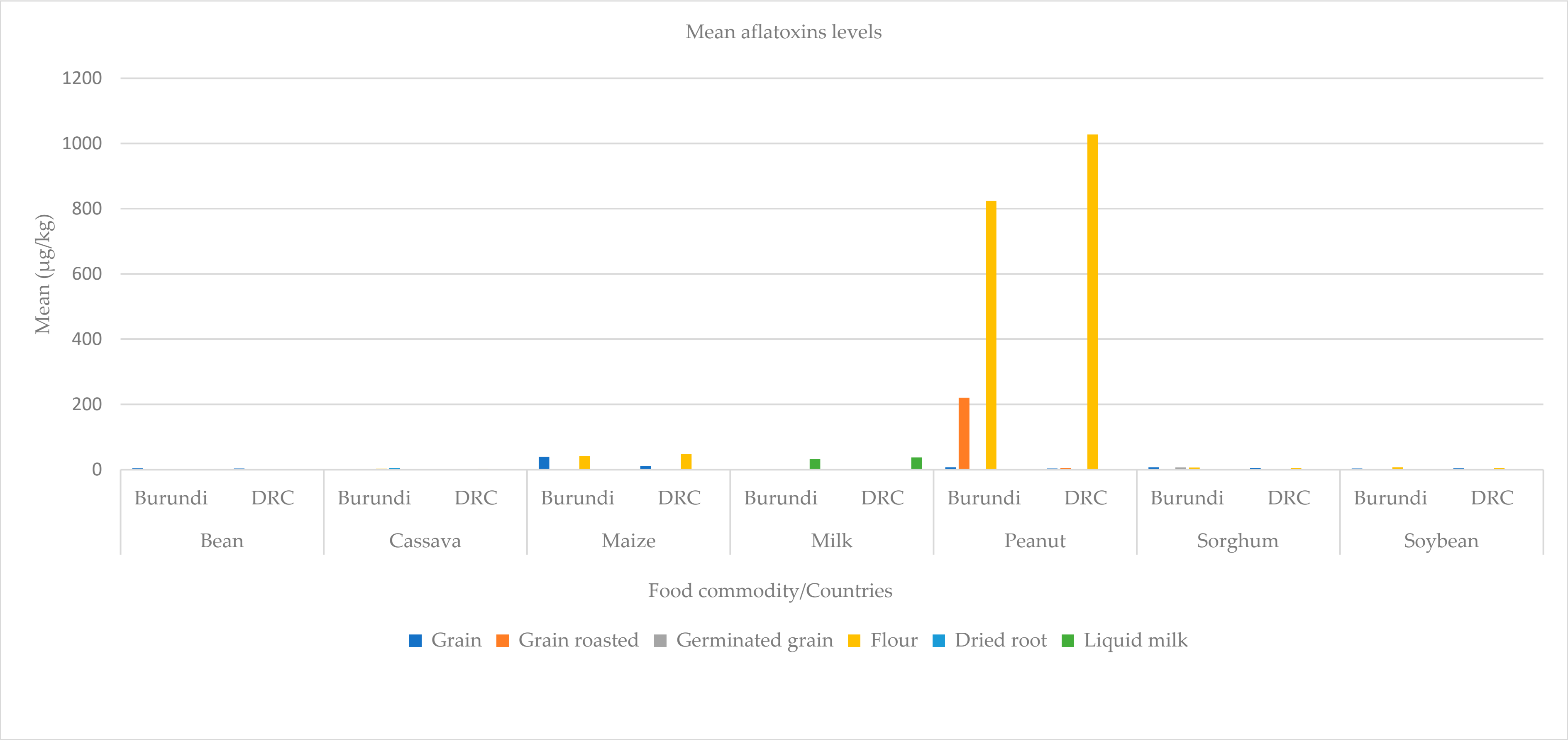

Figure S3. Comparative visualization of AFT levels in selected food commodities (beans, cassava, maize, milk, peanuts, sorghum, and soybean) between the DRC and Burundi, based on data extracted from Udomkun et al. [54]. Values represent reprocessed quantitative data from the original study, harmonized across commodities and matrices (grain, flour, liquid, and derived products) to support cross-country comparison. The figure highlights consistently higher aflatoxin levels in the DRC, particularly in processed products such as flours and peanut-derived products.

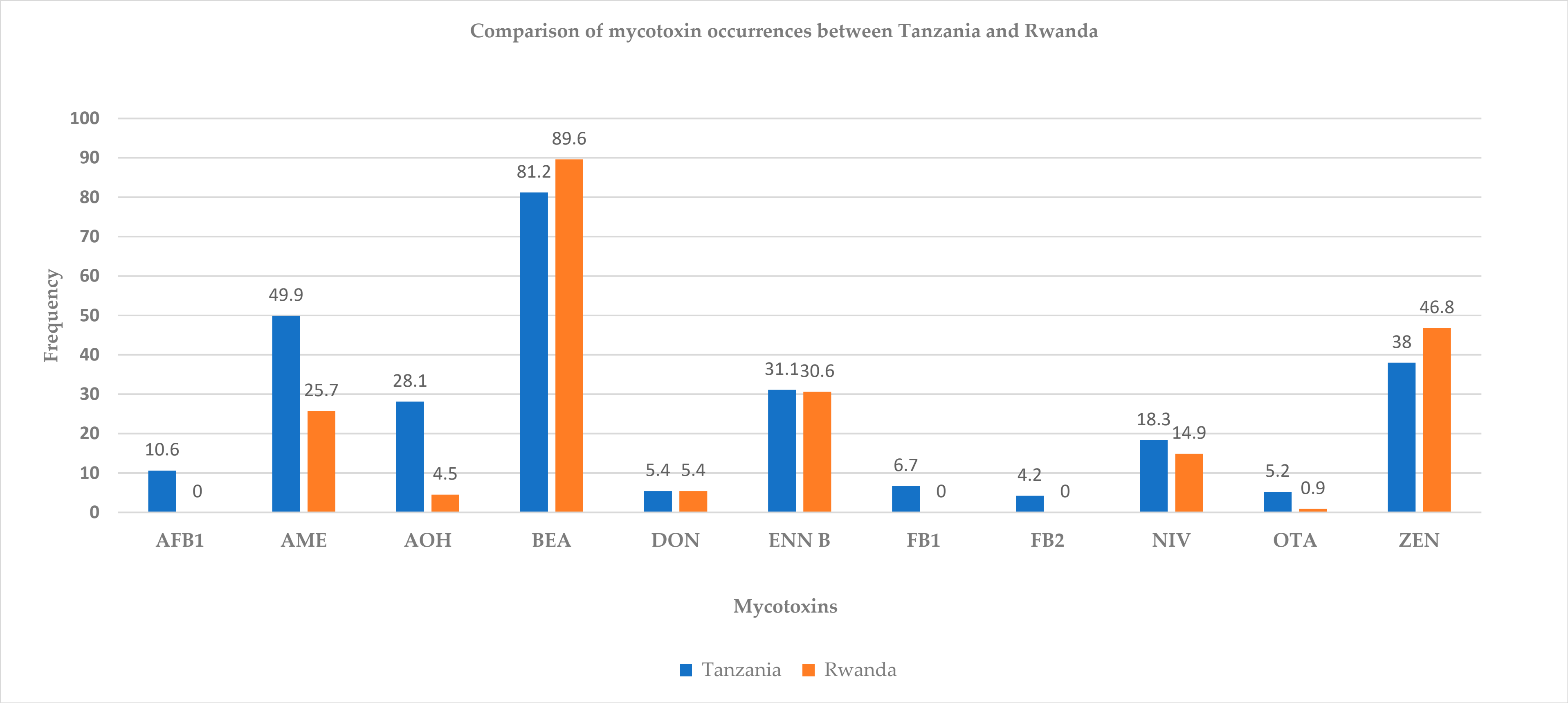

Figure S4. Comparative visualization of the occurrence (%) of multiple mycotoxins (AFB1, AME, AOH, BEA, DON, ENN B, FB1, FB2, NIV, OTA, and ZEN) in selected cassava flour samples from Tanzania and Rwanda, based on data extracted from Sulyok et al. [89]. Values represent reprocessed occurrence data from the original study, harmonized to enable direct comparison between countries. The figure illustrates distinct mycotoxin profiles, with higher prevalence of BEA and ZEN in Rwanda and greater occurrence of AFB1, AME, AOH, and NIV in Tanzania.
